# Endothelial PAR2 activation evokes resistance artery relaxation

**DOI:** 10.1002/jcp.30973

**Published:** 2023-02-15

**Authors:** Xun Zhang, Matthew D. Lee, Charlotte Buckley, Morley D. Hollenberg, Calum Wilson, John G. McCarron

**Affiliations:** ^1^ Strathclyde Institute of Pharmacy and Biomedical Sciences University of Strathclyde Glasgow UK; ^2^ Department of Physiology and Pharmacology and Department of Medicine University of Calgary Cumming School of Medicine Calgary Alberta Canada

**Keywords:** calcium signaling, endothelium, protease activated receptor, resistance artery, vascular relaxation

## Abstract

Protease‐activated receptor‐1 & ‐2 (PAR1 and PAR2) are expressed widely in cardiovascular tissues including endothelial and smooth muscle cells. PAR1 and PAR2 may regulate blood pressure via changes in vascular contraction or relaxation mediated by endothelial Ca^2+^ signaling, but the mechanisms are incompletely understood. By using single‐cell Ca^2+^ imaging across hundreds of endothelial cells in intact blood vessels, we explored PAR‐mediated regulation of blood vessel function using PAR1 and PAR2 activators. We show that PAR2 activation evoked multicellular Ca^2+^ waves that propagated across the endothelium. The PAR2‐evoked Ca^2+^ waves were temporally distinct from those generated by muscarinic receptor activation. PAR2 activated distinct clusters of endothelial cells, and these cells were different from those activated by muscarinic receptor stimulation. These results indicate that distinct cell clusters facilitate spatial segregation of endothelial signal processing. We also demonstrate that PAR2 is a phospholipase C‐coupled receptor that evokes Ca^2+^ release from the IP_3_‐sensitive store in endothelial cells. A physiological consequence of this PAR2 signaling system is endothelium‐dependent relaxation. Conversely, PAR1 activation did not trigger endothelial cell Ca^2+^ signaling nor relax or contract mesenteric arteries. Neither did PAR1 activators alter the response to PAR2 or muscarinic receptor activation. Collectively, these results suggest that endothelial PAR2 but not PAR1 evokes mesenteric artery relaxation by evoking IP_3_‐mediated Ca^2+^ release from the internal store. Sensing mediated by PAR2 receptors is distributed to spatially separated clusters of endothelial cells.

## 1 INTRODUCTION

The endothelium is a monolayer of cells that lines all blood vessels and it regulates blood fluidity, vascular contractility, and vascular permeability (Félétou, 2012). An unresolved question is how the endothelium integrates multiple physiological inputs to regulate vascular responses. A critical step in the endothelial cells' response to various physiological stimuli is the generation of intracellular Ca^2+^ signals (Alexander et al., 2019; McCarron et al., 2019). These Ca^2+^ signals underlie the endothelium's control of numerous physiological processes such as cell proliferation, adhesion, and migration (Clapham, 2007; Hill‐Eubanks et al., 2011).

One class of receptor that detects specific stimuli is the G protein‐coupled protease‐activated receptor (PAR) family. PARs are unique in their lack of physiologically soluble ligands. Instead, PAR receptors are activated by several trypsin‐like proteases that mediate, for example, the cellular effects of thrombin in triggering hemostasis and thrombosis. These proteases cleave N‐terminal peptides that enable tethered ligand activation of PARs (Adams et al., 2011; Hollenberg & Houle, 2005). PARs may also be activated by synthetic peptides that correspond to the first six amino acids of the tethered N‐terminal ligands of the receptor. These peptides activate PARs without a requirement for cleavage of the receptor.

There are four main members of the PAR receptor family that have been identified and all share topological homology (PAR1–4). However, as PAR3 and PAR4 are predominantly expressed outside the vascular wall, our investigations focused on PAR1 and PAR2. PAR1 mediates processes involved in coagulation and in altering cell permeability (Coughlin, 2000; Hill et al., 2020; Vu et al., 1991). PAR2 is proposed to initiate proliferation in human smooth muscle cells (Bono et al., 1997) and inflammation development in the vasculature (Anthoni et al., 2007; Kawabata et al., 1998).

For human PAR1, the protease thrombin cleaves at Arg^41^Serine^42^ at extracellular domains to generate the tethered ligand, S^42^FLLRN. This tethered ligand can then interact with the receptor extracellular domains (Nanevicz et al., 1995; Zhang et al., 2013). The conformational change in PAR1 alters the receptors affinity to intracellular G proteins to mediate downstream signaling (Seeley et al., 2003). Activated protein C (APC) cleaves PAR1 at the same site as thrombin and at an alternative site (Arg^46^ Asn^47^) in endothelial cells (Mosnier et al., 2012). However, APC exerts distinct consequences from thrombin by inhibiting thrombin‐induced transforming protein RhoA signaling and preventing endothelial barrier disruption (Singh et al., 2007). Metalloproteases (MMPs) also activate intracellular signaling pathways by cleaving PAR1, though at different sites from thrombin. MMP‐1 cleaves PAR1 at Asp^39^Pro^40^, leading to activation of the G_α12/13_ pathway and resulting in pro‐inflammatory signaling and cancer invasion (Austin et al., 2013; Boire et al., 2005). Thus, different mechanisms of PAR activation can generate distinct physiological responses.

PAR1 is expressed mainly on platelets, but is also found on vascular endothelial cells, where it can stimulate pro‐inflammatory or anti‐inflammatory signaling depending on the activating protease and the physiological context (De Ceunynck et al., 2018). Typically, thrombin‐induced PAR1 activation causes pro‐inflammatory endothelial signaling. However, cleavage of PAR1 by alternative proteases, such as APC, can protect endothelial cells from inflammatory mediators. As an alternative to proteolytic cleavage, PAR1 is also specifically activated by the synthetic peptide TFLLR‐NH_2_ (TFLLR; Vassallo et al., 1992). While studies have shown that PAR1 activation may modulate endothelial barrier integrity, and limit vascular inflammation (Grimsey & Trejo, 2016), little is known of the physiological consequences of PAR1 activation of endothelial cells in intact blood vessels.

PAR2 is expressed in smooth muscle cells and in endothelial cells. PAR2 activation induces prostate smooth muscle contraction (Paul et al., 2019), vasodilation in isolated rat and porcine arteries (El‐Daly et al., 2014; Villari et al., 2017), tissue factor‐mediated inflammation (Pawlinski et al., 2004), and cancer cell migration (Morris et al., 2006). PAR2 is the only member in the PAR family that is activated by trypsin at Arg^36^Serine^37^ (Heuberger & Schuepbach, 2019). PAR2 may also be activated by a variety of other extracellular proteases that include tissue kallikreins, coagulation proteases (VIIa, Xa), leukocyte proteases (tryptase), and transmembrane proteases (Rothmeier & Ruf, 2012). In various cell lines, PAR2 stimulation triggers G protein signaling pathways that include G_q_‐mediated increases in intracellular Ca^2+^ (Corvera et al., 1997) and G_12/13_‐mediated nuclear factorκB (NFκB) pathway activation (Kanke et al., 2001). G protein‐independent signaling via the β‐arrestin pathway may also occur via PAR2 activation (DeFea et al., 2000). PAR2 may also be specifically activated by the synthetic peptide 2‐furoyl‐LIGRLO‐amide (2fLI) in the absence of enzymatic cleavage of the receptor (McGuire et al., 2004); for example, injection of PAR2‐activating peptides evokes hypotension in rats (Cicala et al., 1999). Although the impact of PAR activation on rat and mouse conduit vessel preparations has been studied, the effects on intact resistance‐sized arteries (key sites of blood pressure regulation) have never been evaluated in depth.

PAR1 and PAR2 have each been implicated in blood pressure regulation and development of hypertension (Capers et al., 1997; Cicala, 2003; Moffatt & Cocks, 1998; Trottier et al., 2002). However, although PAR1 and PAR2 may evoke significant cardiovascular effects, there is uncertainty on the dominant functional receptor, and the role of endothelial Ca^2+^ signaling in PAR‐mediated regulation of vascular contractility in mesenteric resistance vessels. In this study, we investigated the physiological functions and control of endothelial Ca^2+^, evoked by PAR1 and PAR2 in isolated rat mesentery, using specific PAR receptor activating peptides (TFLLR and 2fLI) and the proteases, thrombin and trypsin.

## 2 MATERIALS AND METHODS

### 2.1 Animals

All animal care and experimental procedures were carried out with the approval of the University of Strathclyde Animal Welfare and Ethical Review Board (Schedule 1 procedure; Animals [Scientific Procedures] Act 1986, UK), under UK Home Office regulations. All procedures were in accordance with the regulation of the University of Strathclyde Animal Welfare and Ethical Review Body. Adult male SD (Sprague‐Dawley) IGS rats (10–12 weeks old) were used in this study. All animals were housed three per cage (RC2F cages, North Kent Plastics Company) in the enriched environment with aspen wood chew sticks, hanging huts, and nesting materials (Sizzle nest, LBS Technology). All animals had access to fresh water and chow (RM1, Special Diet Service) ad libitum. Animals were housed at room temperature (19–23°C, set point 21°C), humidity 45%–65%, and a 12‐h light cycle. Animals were euthanatized by cervical dislocation and the whole mesentery was dissected out and transferred immediately to a physiological salt solution (PSS) of the following composition (in mM): 145.0 NaCl, 2.0 MOPS (3‐(*N*‐morpholino) propanesulfonic acid, 4‐morpholinepropanesulfonic acid), 4.7 KCl, 1.2 NaH_2_PO_4_, 5.0 Glucose, 0.02 EDTA (ethylenediaminetetraacetic acid), 1.17 MgCl_2_, 2.0 CaCl_2_ (pH adjusted to 7.4 with NaOH).

### 2.2 Chemicals

The PAR activating peptides (>95% pure by HPLC) TFLLR‐NH_2_ (PAR1) and 2‐furoyl‐LIGRLO‐amide (PAR2) were synthesized in the University of Calgary peptide synthesis facility. Acetylcholine (A6625), 2‐APB (D9754), Caffeine (C0750), phospholipase C (PLC) inhibitor—U73122 (U6756), TRPV4 blocker—HC 067047, CPA (C1530), phenylephrine (P6126), sodium nitroprusside (S0501), trypsin (T1426), and thrombin (T4648) were from Sigma UK. The stock solution of acetylcholine (ACh, 100 mM) and phenylephrine (10 mM) was prepared in MilliQ water. Sodium nitroprusside and caffeine were dissolved in PSS. 2‐APB and U73122 were dissolved in dimethyl sulfoxide (DMSO, D8418) and diluted in PSS to the final concentration. Cal‐520/AM (ab171868) was obtained from Abcam (UK). Pluronic F‐127 was obtained from SiChem (Germany).

### 2.3 Tissue preparation

The isolated mesentery was pinned in a Petri dish coated with Sylgard silicone (Dow Chemical Company) using 0.2 mm pins (Austerlitz) and second‐order mesenteric arteries were dissected and gently cleaned of connective tissue and fat. Arteries were then cut open longitudinally using micro‐scissors and pinned flat (endothelium facing upwards; en face preparation) on the bottom of a custom‐made Sylgard‐coated bath chamber.

### 2.4 Endothelial cell Ca^2+^ imaging

The endothelium was loaded preferentially with the acetoxymethyl ester form of the Ca^2+^ indicator, Cal520‐AM (5 µM with 0.02% pluronic F‐127 in PSS at 37°C for 30 min) (Wilson et al., 2016). After loading, the preparation was gently washed with PSS to remove excess dye before imaging. The bath chamber was fixed on a custom‐made chamber holder and fitted on the stage of an upright fluorescence microscope (FN‐1, Nikon) equipped with a 40× water immersion objective lens (0.8 numerical aperture; Nikon) and a back‐illuminated electron‐multiplying charge‐coupled device (EMCCD) camera (1024 × 1024 13 µm pixels; iXon 888, Andor) for visualization of Ca^2+^ activity at 10 Hz. Fluorescence excitation (488 nM wavelength for Cal520) was supplied by a CoolLED pE‐4000 (CoolLED).

When measuring diameter changes, the artery was imaged with a 16× water immersion objective lens (0.8 numerical aperture; Nikon). The imaging system was controlled and Ca^2+^ images were recorded using the open‐source microscopy software, Micro‐Manager (Edelstein et al., 2010, 2014).

### 2.5 Ca^2+^ imaging protocols

Endothelial Ca^2+^ signaling was examined in response to muscarinic and PAR receptor activation. In all experiments, ACh (100 nM) was perfused into the bath before each experiment to confirm endothelial cell viability. Preparations were then washed with PSS for 10 min, after ACh application, and allowed to equilibrate. Following confirmation of cell viability, and unless otherwise indicated, the effects of various agonists and antagonists on endothelial Ca^2+^ activity were studied using a paired experimental approach (i.e., before vs. after receptor blockade on the same preparation). Incubation times for each intervention are indicated in the text.

In all experiments, agonists were perfused into the bath and the evoked Ca^2+^ response was recorded for 10 min followed by a 10 min wash with PSS and 10 min rest before additional experimental manipulation. In all experiments, a stable baseline was attained before the subsequent addition of an agonist. In experiments designed to examine the concentration dependence of the various activators, full noncumulative concentration responses were obtained in each en face preparation. This was achieved by sequentially applying each of the agonists at various concentrations (0.5–100 nM). After each agonist application, arteries were washed with PSS for 10 min and allowed to re‐equilibrate. Only one concentration–response curve was generated for each mesenteric artery preparation.

For subsequent experiments, the PAR1‐activating peptide, TFLLR‐NH_2_, was used at a concentration selective for PAR1 activation (10 μM). Concentrations greater than 10 μM may also activate PAR2 (Hollenberg et al., 1997). Thrombin, which selectively activates PAR1, was also used at a concentration of 0.2 U/ml (2 nM), which does not activate PAR2. The PAR2‐activating peptide, 2‐furoyl‐LIGRO‐NH_2_ (2fLI), was used at a concentration of 100 nM, which potently activates PAR2, but does not activate PAR1. Trypsin was used at low concentrations (0.2 U/ml; 0.4 nM) that selectively activate PAR2, but not PAR1 (Hollenberg et al., 1997).

### 2.6 Ca^2+^ imaging analysis

Single‐cell Ca^2+^ signals were extracted and analyzed by using custom‐written python software (Wilson et al., 2016). Circular regions of interest (ROI) were generated for each cell (radius = 10 µM), and each ROI was assigned a unique identification number. Subtle shifts of ROIs between each recording were corrected using an ImageJ Plug‐in. The Ca^2+^ signal for each ROI was extracted by averaging the fluorescence values expressed as ratios (*F*/*F*_0_) of fluorescence counts (*F*) against baseline values (*F*_0_) in resting conditions. Baseline was identified as the fluorescence intensity occurring during the lowest background noise during 100 frames (10 s) of recording before the introduction of agonists or antagonists. The amplitude and number of oscillations from each cell were extracted and aligned to the baseline values. Active cells were identified using a zero‐crossing peak‐detection algorithm as cells in which the fluorescence signals exceeded five times the SD of baseline noise (Wilson et al., 2016). To describe the time course of the onset, and steady‐state responses to activation, responses were measured at two time points. The first (*t*1) was 1 min after the defined baseline measurement, while the second (*t*2) was 6 min after baseline measurement.

### 2.7 Assessment of vascular reactivity

Changes in vascular reactivity were assessed in isolated mesenteric arteries (~150 µm diameter) mounted en face and prepared as described above. Arteries were visualized at 5 Hz using a 16× magnification, 0.8 numerical aperture objective. The resulting 832 × 832 µm field of view allowed quantification of vascular reactivity using VasoTracker edge‐detection algorithms (Lawton et al., 2019). In these experiments, arteries were precontracted with phenylephrine (1 µM). When the contraction stabilized, ACh (100 nM) was added to the bath to evoke endothelium‐dependent relaxation. After a 20 min recording, the preparation was washed with PSS for 20 min and allowed to rest for a further 20 min. The same preparation was then contracted again (as above) and vascular reactivity to 2fLI (100 nM) was assessed.

The endothelial contribution to 2fLI‐evoked control of blood vessel contractility was determined by removing the endothelial layer using a human hair (Aoqui et al., 2014). In these experiments, control responses were obtained and then the endothelium was removed and vascular reactivity reassessed in the same artery. Sodium nitroprusside (100 µM) was applied at the end of each experiment to confirm smooth muscle cell viability.

### 2.8 Statistical analysis

Summarized data are presented as mean ± SD of *n* biological replicates. Data were analyzed using two‐tailed Student's *t‐*test (paired data) or two‐tailed one‐way ANOVA with Tukey's multiple‐comparisons test, as indicated. Concentration–response curves were analyzed with a log (agonist) versus response algorithm to define EC_25_ and EC_100_. *A p* < 0.05 was defined as statistically significant. All statistical analyses were performed using GraphPad Prism version 8.0 (GraphPad Software).

## 3 RESULTS

### 3.1 PAR2 endothelial Ca^2+^ signaling

**Figure 1:**
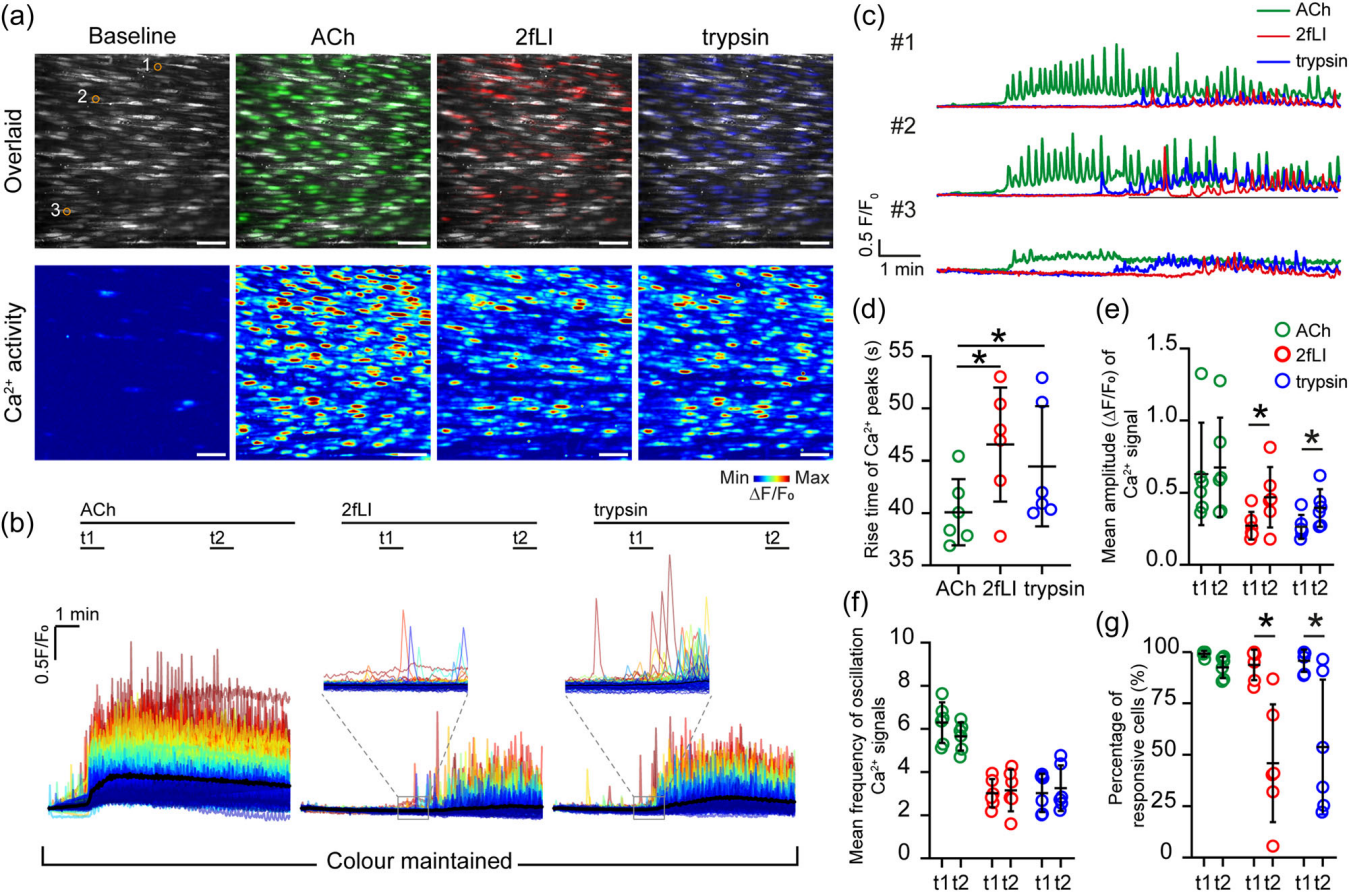
Protease activated receptor 2 (PAR2) stimulates different profiles of Ca^2+^ signaling from muscarinic receptor activation. (a) Overlaid images (top) of raw and pseudocolored Ca^2+^ signals and (bottom) heat map images showing maximum intensity projections (∆*F*/*F*_0_) of Ca^2+^ signals evoked by ACh, 2fLI, and trypsin during 10 min recordings. Scale bar = 50 µm. (b) Colored overlaid Ca^2+^ signaling traces extracted from each cell shown in (a) according to the intensity of signals (*F*/*F*_0_, from blue, low to red, high). The black line shows the averaged Ca^2+^ signal in response to ACh (left), 2fLI (middle), and trypsin (right). (c) Example single Ca^2+^ signal traces in response to ACh, 2fLI, and trypsin from cells indicated in (a, top left panel). (d) Averaged rise time of Ca^2+^ peaks, (e) mean peak amplitude, (f) frequency of Ca^2+^ oscillation evoked by ACh, 2fLI, and trypsin at *t*1 (first min after defined baseline) and *t*2 (sixth min after defined baseline) and (g) mean percentage of active cells evoked by ACh, 2fLI, and trypsin at *t*1 and *t*2. For all summary data (d–g), *n* = 6, *p < 0.05.

To examine endothelial regulation by PAR2 activation, intracellular Ca^2+^ signals in response to the PAR2 agonists, 2‐Furoyl‐LIGRLO‐amide (2fLI, 100 nM) and trypsin (0.2 U mL^−1^), were each measured. 2fLI (100 nM) and trypsin (0.2 U ml^−1^) each evoked endothelial Ca^2+^ signals that propagated across cells (Ca^2+^ waves). The Ca^2+^ signals evoked by 2fLI and trypsin increased slowly in amplitude (Figure 1a–g), then remained elevated above baseline levels while the agonists were present. 2fLI‐evoked Ca^2+^ signals were abolished by the selective PAR2 inhibitor, AZ 3451 (Supporting Information: Figure S1).

### 3.2 Spatial and temporal signaling heterogeneity

Interestingly, activation of the muscarinic G protein‐coupled receptor with ACh (100 nM) generated a significantly different profile of Ca^2+^ responses to those of either 2fLI or trypsin. ACh‐evoked Ca^2+^ responses increased sharply in amplitude, followed by sustained Ca^2+^ oscillations (Figure 1c and Supporting Information Figure S2). The different profiles of Ca^2+^ response mediated by protease activated receptor 2 (PAR2) and muscarinic receptor activation are consistent with distinct physiological roles in the native endothelium.

**Figure 2:**
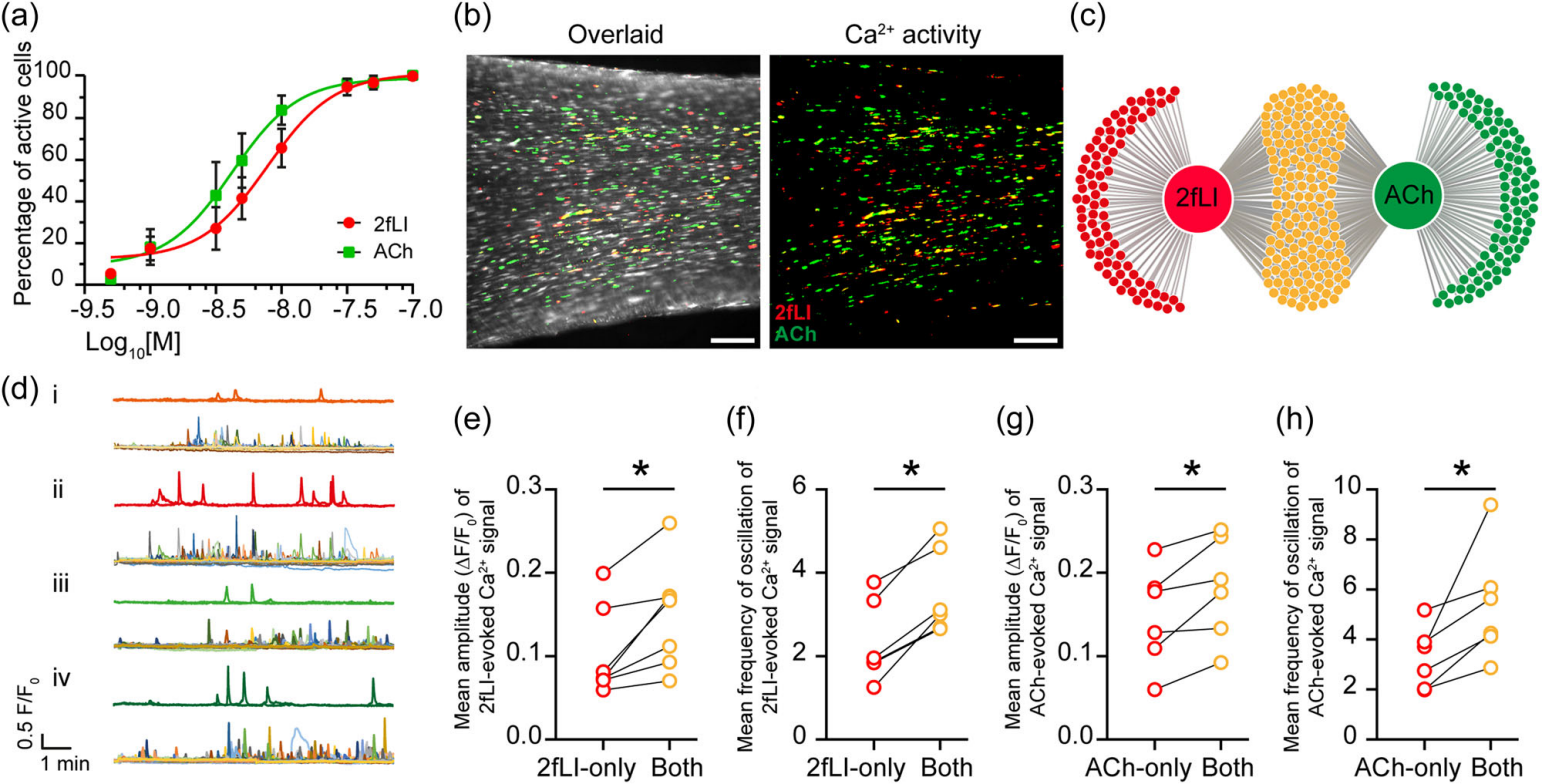
Heterogeneity of 2fLI‐ and ACh‐evoked Ca^2+^ signaling. (a) Concentration response (% of cells activated) by ACh (green) or 2fLI (red). (b) Pseudo‐colored images of cells responding to ACh (green; 3 nM) and 2fLI (red; 5 nM). Cells activated by both agonists are in yellow. Scale bar = 50 µm. (c) Venn diagram showing summary data of cells activated by ACh and 2fLI in (b). Red dots represent cells activated by 2fLI only; the green dots represent cells activated by ACh only. Yellow dots show the cells activated by 2fLI and ACh. (d) Ca^2+^ traces from cells that responded only to 2fLI (i) or 2fLI evoked Ca^2+^ signals in cells that responded to 2fLI and ACh (ii) or ACh evoked Ca^2+^ signals from cells that respond only to ACh (iii) or ACh evoked Ca^2+^ signals from cells that respond to 2fLI and ACh (iv). In each case the lower panel shows all cells that responded and the upper trace plots two representative examples. (e) Mean amplitude of 2fLI‐evoked Ca^2+^ increase and (f) frequency of Ca^2+^ signal oscillations from cells that respond only to 2fLI and respond to ACh and 2fLI. (g) Mean amplitude of ACh‐evoked Ca^2+^ signals and (h) frequency of Ca^2+^ signal oscillations from cells that only respond to ACh or that respond to ACh and 2fLI. *n* = 6, *p < 0.05. Tissues were activated by ACh and 2fLI on two consecutive occasions at EC_25_ concentration with 10 min wash and 10 min rest in between.

Given the differences in intracellular Ca^2+^ responses evoked by PAR2 and muscarinic receptor activation, a series of experiments were undertaken to explore heterogeneity in the responses to the two G protein‐coupled receptors. To examine the sensitivity of the endothelium to PAR2 or muscarinic receptor activation, equivalent concentrations of each agonist were first determined (25% effective concentration [EC_25_]). To do this, full noncumulative concentration response experiments were carried out for both ACh and 2fLI (Figure 2a). The EC_25_ was 2.084 nM (95% CI from 0.618 to 3.058 nM) for ACh and 3.627 nM (95% CI from 2.121 to 5.008 nM) for 2fLI. The EC_25_ of each agonist was used to explore the heterogeneous behavior in endothelial cells to PAR2 and muscarinic receptor activation.

The EC_25_ concentration of ACh and 2fLI were perfused separately onto the same preparation (10 min wash and 10 min rest between each activation) (Zhang et al., 2019). Overlaid images show the separation and overlap (yellow) of cells activated by ACh (green) and 2fLI (red) at the EC_25_ concentrations (Figure 2b). In these experiments, 66% of the cells that were activated by the EC_25_ concentrations of 2fLI also responded to ACh, and 64% of the cells that were activated by ACh also responded to 2fLI. The remaining 35% of cells responded only to either ACh or 2fLI (Figure 2c).

The Ca^2+^ response also varied within different subgroups of cells. In cells that responded to ACh and 2fLI the amplitude of 2fLI‐evoked Ca^2+^ responses were larger, and oscillation frequency greater, than those from the cells that respond to 2fLI alone. This also occurred in cells that responded to ACh (Figure 2d–h). These observations suggest that cells not only have different populations of receptors expressed but also that receptor coupling with Ca^2+^ signaling differs in the various populations of cells.

### 3.3 Ca^2+^ release and Ca^2+^ entry in PAR2‐evoked endothelial Ca^2+^ signals

Endothelial Ca^2+^ responses may be generated by either influx across the plasma membrane, or release from the intracellular store, or both. To investigate the mechanisms involved in PAR2‐mediated endothelial Ca^2+^ signaling, PAR2‐medated responses were examined after the removal of extracellular Ca^2+^ or block of Ca^2+^ entry via TRPV4.

**Figure 3:**
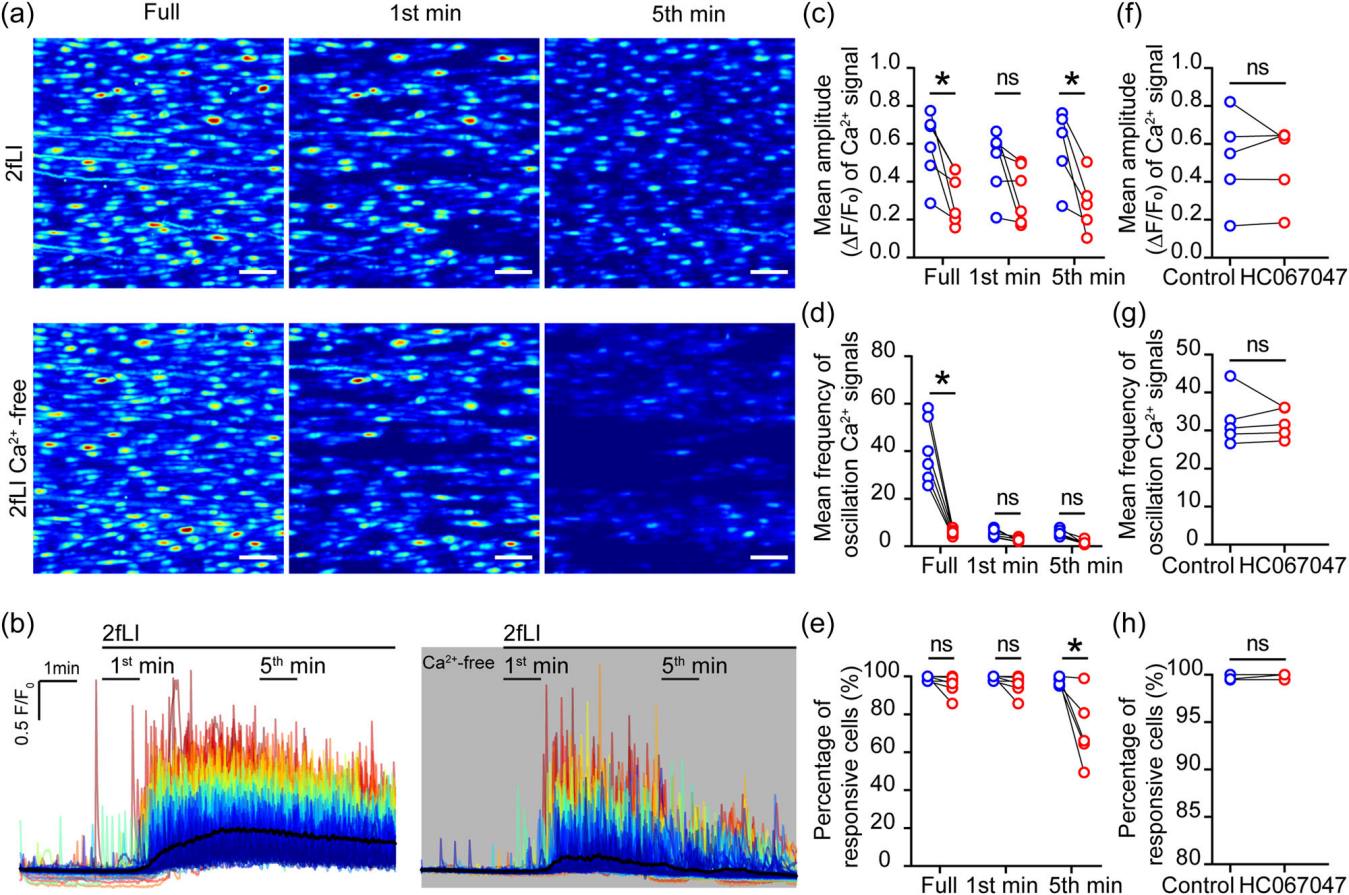
Protease activated receptor 2 (PAR2) evoked Ca^2+^ signals do not require Ca^2+^ entry. Endothelial cells were activated by 2fLI in PSS, followed by 10 min wash and 10 min rest. The same preparation was activated again by 2fLI in Ca^2+^‐free PSS. (a) Heat map images show maximum intensity projection (∆*F*/*F*_0_) of Ca^2+^ signals evoked by 2fLI at the first and fifth minutes after defined baseline in a 10 min recording. (b) Colored overlaid Ca^2+^ signaling traces extracted from each individual cell shown in (a) according to the intensity of signals. (c) Summarized mean peak amplitude of Ca^2+^ signaling, (d) Number of Ca^2+^ oscillation evoked by 2fLI and (e) percentage of 2fLI responsive cells during the first and fifth minute after defined baseline. Effect of a TRPV4 blocker. The endothelium was activated by 2fLI, followed by 10 min wash and 10 min rest. The artery was then incubated with selective TRPV4 blocker, HC 067047 (10 µM) for 10 min. The same artery was activated again by 2fLI in the presence of HC 067047. (f) Summarized mean peak amplitude of Ca^2+^ signals, (g) frequency of Ca^2+^ oscillation evoked by 2fLI and (h) percentage of 2fLI responsive cells. For all summary data (c–h), *n* = 5, *p < 0.05.

2fLI‐evoked (100 nM) Ca^2+^ responses persisted after block of Ca^2+^ entry via TRPV4 by HC 067047 (10 µM) or in the absence of external Ca^2+^ (Figure 3a–e). However, in contrast to the maintained responses obtained in the presence of external Ca^2+^, mean Ca^2+^ levels returned to baseline levels after ∼5 min of stimulation as the internal store was depleted. The preservation of the initial response, despite the absence of external Ca^2+^, suggests that the primary response may occur via Ca^2+^ release from the IP_3_‐sensitive internal store.

**Figure 4:**
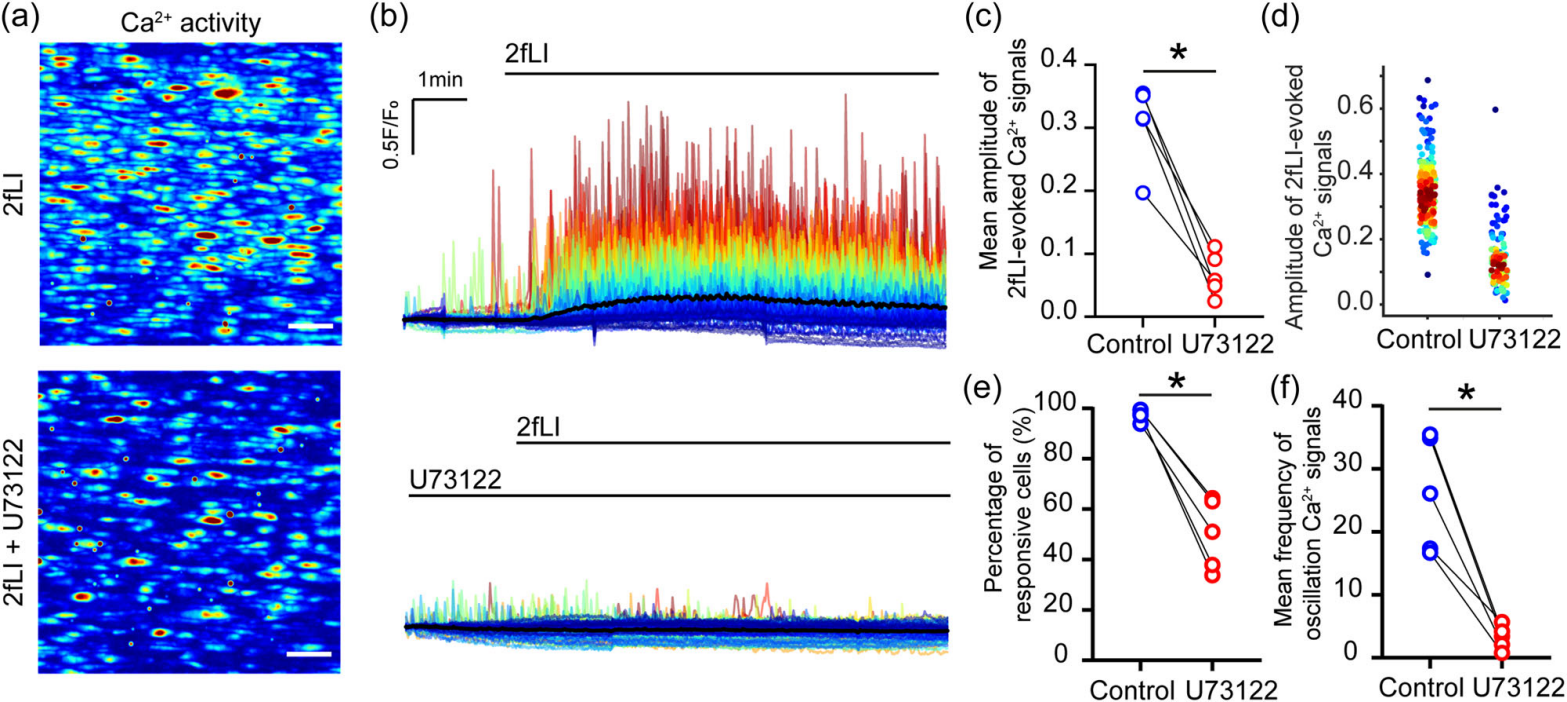
Protease activated receptor 2 (PAR2) is a phospholipase C (PLC)‐coupled receptor. (a) Heat map images showing maximum intensity projection (∆*F*/*F*_0_) of Ca^2+^ signals evoked by 2fLI and 2fLI with the PLC inhibitor U73122. Scale bar = 50 µm. (b) Colored overlaid Ca^2+^ signaling traces extracted from each individual cell shown in (a) according to the intensity of signals. (c) Summarized mean peak amplitude of Ca^2+^ signaling evoked by 2fLI and 2fLI and U73122. (d) Density plot of peak value of Ca^2+^ signaling from each cell in (a). Individual data points have been colored according to the density of particular values (blue, low to red, high). ((e) Summarized percentage of 2fLI responsive cells. (f) Summarized frequency of Ca^2+^ oscillation evoked by 2fLI and 2fLI + U73122. For all summary data (c, d, e, f) *n* = 5, *p < 0.05.

**Figure 5:**
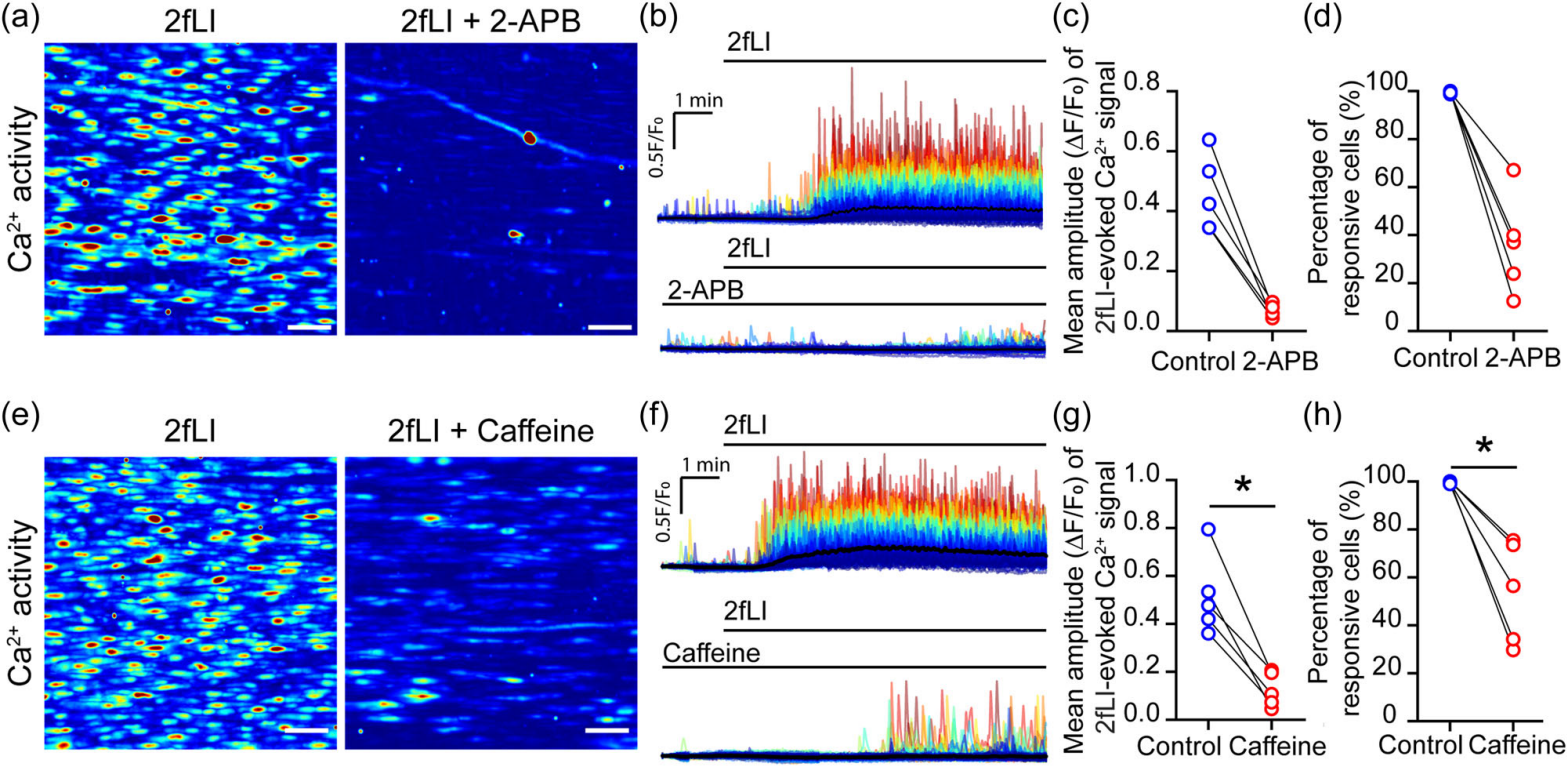
Protease activated receptor 2 (PAR2) mediates Ca^2+^ release via the IP_3_ receptor. (a) Heat map images showing maximum intensity projections (∆*F*/*F*_0_) of Ca^2+^ signals evoked by 2fLI and 2fLI with 2‐APB. Scale bar = 50 µm. (b) Colored overlaid Ca^2+^ signaling traces extracted from each individual cell shown in (a) according to the intensity of signals. (c) Summarized mean peak amplitude of Ca^2+^ signaling and (d) percentage of 2fLI responsive cells. In other experiments, the effect of the IP_3_R blocker caffeine (10 mM; 10 min) was examined. (e) Heat map images showing maximum intensity (∆*F*/*F*_0_) of Ca^2+^ signals evoked by 2fLI and 2fLI with caffeine. Scale bar = 50 µm. (f) Colored overlaid Ca^2+^ signals extracted from each individual cell shown in (e) plotted according to the intensity of signals. (g) Summarized mean peak amplitude of Ca^2+^ signaling and (H) percentage of 2fLI responsive cells evoked by 2fLI and 2fLI with caffeine. For all summary data (c, d, g, h), *n* = 5, *p < 0.05.

In support, in smooth muscle cells, PAR2 activates phospholipase C (PLC) (Ha et al., 2017). We used a series of pharmacological tools and a paired (i.e., before and after on the same artery) experimental protocol to determine if the PLC–IP_3_–IP_3_R pathway mediates the endothelial Ca^2+^ release to PAR2 stimulation. First, PAR2‐mediated responses were examined before and after PLC inhibition by U73122 (2 µM). U73122 significantly reduced the endothelial Ca^2+^ response to 2fLI (Figure 4a–f). Next, the effects of the IP_3_R blockers, 2‐aminoethoxydiphenyl borate (2‐APB) and caffeine (a potent IP_3_ receptor blocker; Echeverri et al., 2010; Ehrlich et al., 1994; Saleem et al., 2014), were examined. Using two different protocols, IP_3_ receptor inhibition prevents 2fLI‐evoked endothelial Ca^2+^ responses. First, 2‐APB inhibited Ca^2+^ signals to 2fLI (Figure 5a,b). The mean amplitude of Ca^2+^ signals and percentage of active cells were all reduced by 2‐APB (Figure 5c,d). Ca^2+^ signals were also inhibited by caffeine (Figure 5e–h).

**Figure 6:**
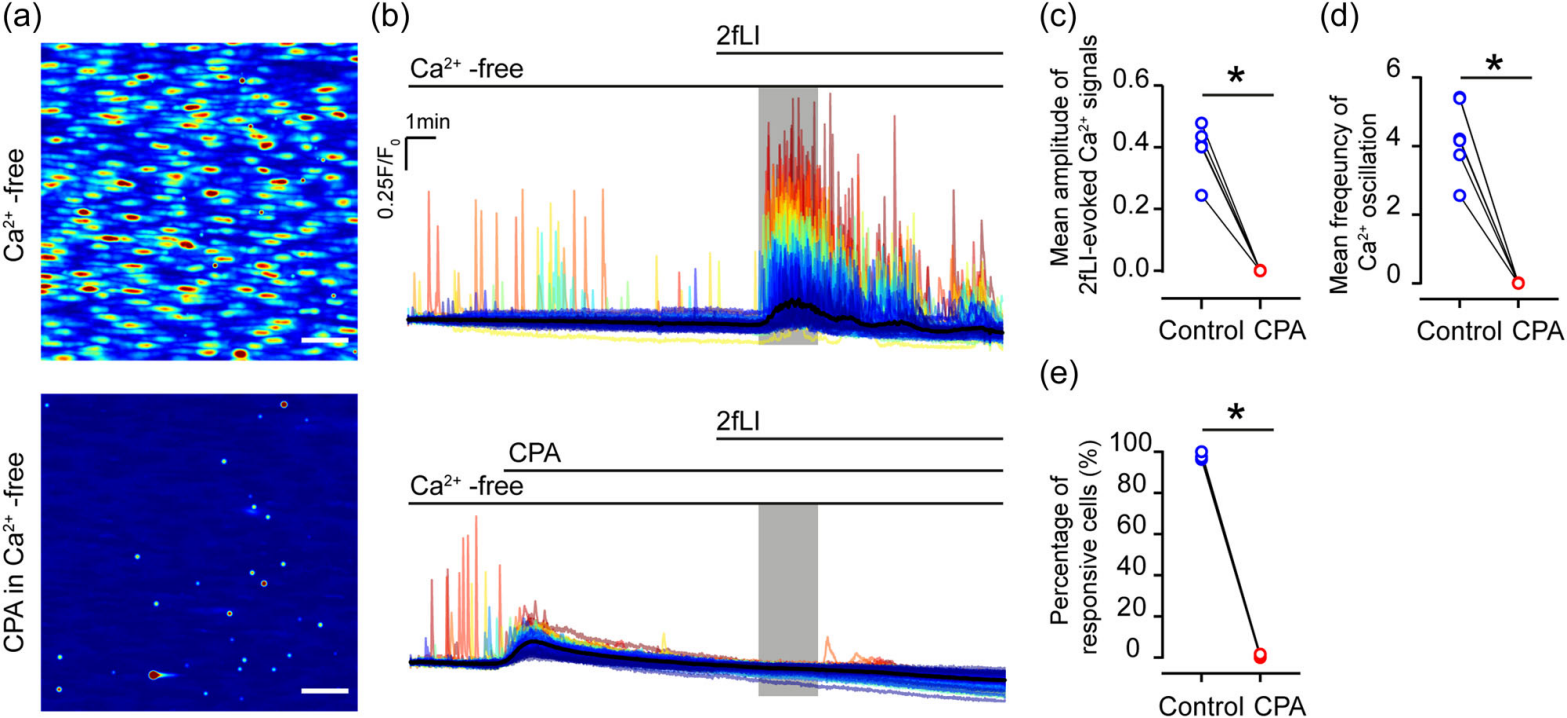
Protease activated receptor 2 (PAR2) evokes Ca^2+^ release from the internal store. (a) Heat map images showing Ca^2+^ activities evoked by 2fLI in the 10th min of recording in the  absence (top) and presence (bottom) of CPA. (b) Colored overlaid Ca^2+^ signaling traces from each cell show the intensity of Ca^2+^ signals (*F*/*F*_0_, from blue, low to red, high) in (a). (c) Summarized data of the mean amplitude of Ca^2+^ signaling, (d) frequency of Ca^2+^ oscillations evoked by 2fLI in each recording, and (e) percentage of 2fLI responsive cells in 1 min duration (gray box) after defined baseline. For all summary data (c–e), *n* = 6, *p < 0.05.

Finally, Ca^2+^ release (in the absence of external Ca^2+^) was examined after the Ca^2+^ store uptake‐leak balance was disrupted using the Ca^2+^ pump inhibitor, cyclopiazonic acid (CPA). When the internal store was depleted by CPA, 2fLI failed to evoke endothelial Ca^2+^ responses (Figure 6a–e). These findings demonstrate that PAR2 are PLC‐coupled receptors that mediate endothelial Ca^2+^ release from the endoplasmic reticulum via the IP_3_ receptor.

### 3.4 PAR1 does not regulate endothelial Ca^2+^ signals

Although PAR1 activation has been reported to regulate intracellular Ca^2+^ levels in cultured endothelial cells (Garcia et al., 1993), we found no evidence that PAR1 evokes endothelial Ca^2+^ signals in intact mesenteric arteries. Indeed, high concentrations of either the PAR1 agonist thrombin (0.2 U ml^−1^), or the PAR1‐activating peptide, TFLLR (10 µM) (Höcherl et al., 2011; O'Loughlin et al., 2005), failed to evoke Ca^2+^ responses in intact artery endothelial cells (Supporting Information: Figure S2), or in freshly isolated sheets of endothelial cells which lack contact with smooth muscle cells (Supporting Information: Figure S3).

As PAR1 and PAR2 may form dimers (Lin et al., 2013), we examined if PAR1 activation modulates the PAR2‐evoked Ca^2+^ responses. PAR2 mediated Ca^2+^ responses were unaffected by PAR1 activation (Supporting Information: Figure S4). Collectively, these results suggest that PAR2, but not PAR1, regulates endothelial cell function in intact mesenteric blood vessels.

### 3.5 PAR regulates endothelial cell control of vascular contractility

**Figure 7:**
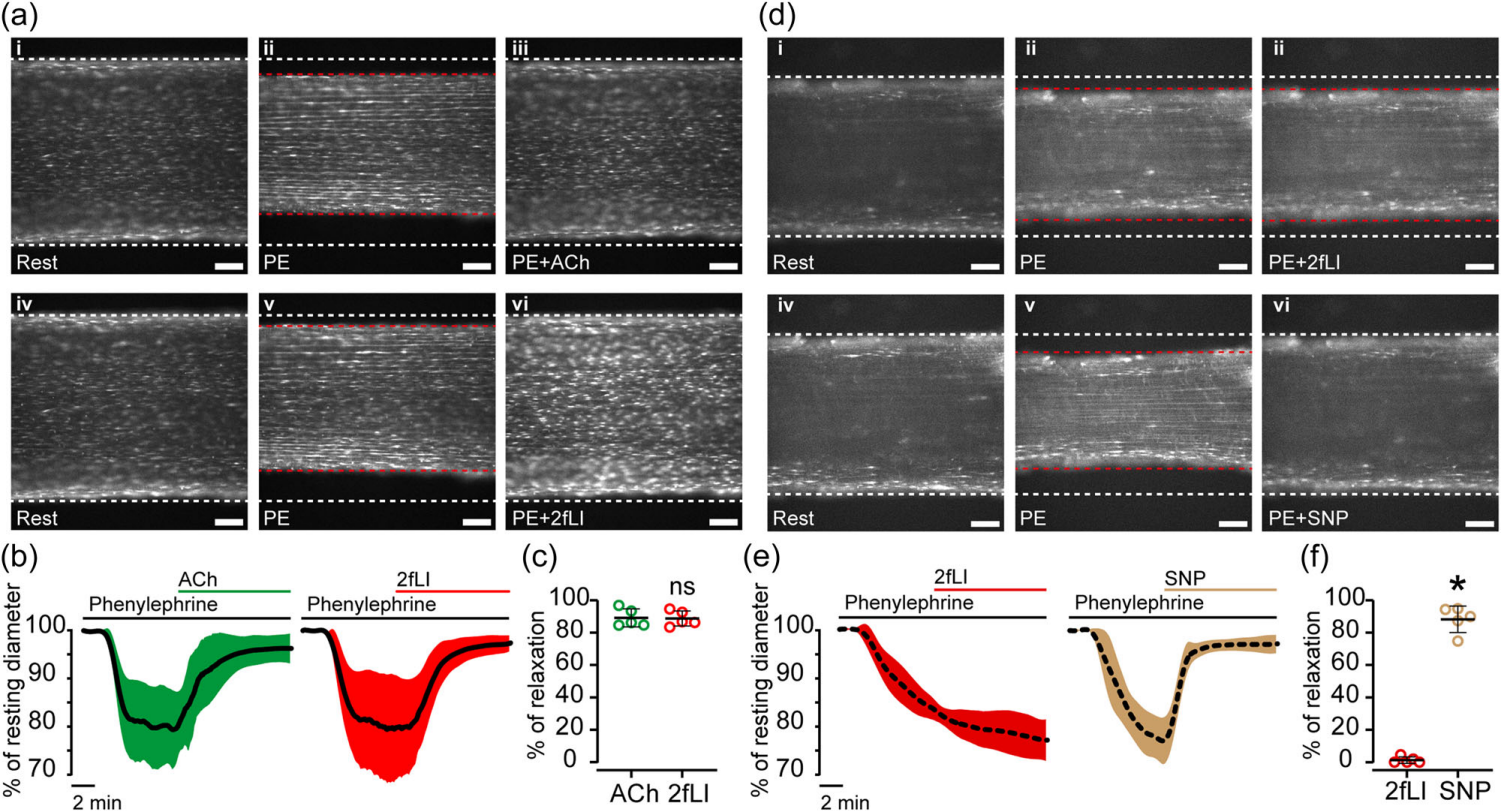
2fLI mediated endothelium‐dependent vasodilation. (a) Raw images of an opened mesenteric artery (same artery throughout) showing perimeter changes. i, resting status; ii, contracted by phenylephrine (1 µM); iii, dilated by ACh (100 nM); iv, resting status; v, contracted by phenylephrine; vi, dilated by 2fLI (100 nM). Scale bar = 200 µm. (b) Plots of diameter changes (%) relative to resting status in 20 min recordings. The solid black line shows the average diameter. The green (ACh) and red (2fLI) filling shows the SD  from eight experiments. (d) Raw images of the same mesenteric artery after endothelium removal (note the absence of cells). i, resting status; ii, contracted by phenylephrine (1 µM); iii, after 2fLI (100 nM); iv, resting status; v, contracted by phenylephrine; vi, after sodium nitroprusside (100 µM). Scale bar = 200 µm. (e) Plots of diameter change (%) relative to resting status in 20 min recordings. The dashed black line shows the average (percentage) resting diameter. The orange (2fLI) and green (sodium nitroprusside) filling represent the SD. Summarized data of percentage of relaxation by ACh and 2fLI in intact arteries (c) and by 2fLI and SNP in endothelium‐denuded arteries (f). For summary data (c, f), *n* = 8, *p < 0.05. ACh, acetylcholine; SNP, sodium nitroprusside.

Endothelial Ca^2+^ levels regulate blood vessel contractility by promoting the production of endothelium‐derived relaxation factors. Thus, we speculated that PAR‐mediated Ca^2+^ responses would manifest as endothelium‐dependent relaxation. To test this hypothesis, mesenteric arteries were preconstricted with phenylephrine (PE, 1 µM) and, after the contraction was stable, relaxed by ACh (100 nM). Arteries were then contracted again (PE; 1 µM) and vascular reactivity to PAR1 and PAR2 activators examined. 2fLI‐evoked a vasodilation of comparable magnitude to ACh (>95% of maximum; Figure 7a–c). This vasodilation was dependent on an intact endothelial cell layer as 2fLI (100 nM) failed to dilate preconstricted, endothelium‐denuded arteries (Figure 7d–f). These arteries relaxed to the endothelium‐independent vasodilator, sodium nitroprusside (SNP, 100 µM).

Neither PAR1 nor PAR2 activation evoked smooth muscle contraction (Figure S5). Collectively, these results demonstrate that PAR2 mediates endothelium‐dependent vasodilation.

## 4 DISCUSSION

Our study represents the first evaluation of PAR regulation of endothelial signaling pathways and contractility in mesenteric resistance arteries. In contrast to previous findings on conduit arteries (intact aorta‐derived rings; Laniyonu & Hollenberg, 1995), the present results suggest that PAR2, rather than PAR1, is the major regulator of endothelial Ca^2+^ signaling and artery dilation. Thus, the intrinsic PAR responses appear to differ in resistance versus capacitance vessels. Our results also show that PAR2 evokes endothelial‐dependent vasodilation as a result of IP_3_‐mediated Ca^2+^ release. Conversely, PAR1 activation does not evoke endothelial Ca^2+^ signals nor does it regulate vascular contractility in mesenteric arteries. These data differ from the actions of PAR1 activation in isolated aorta ring and coronary artery preparations, where thrombin and the PAR1‐activating peptide each cause an endothelium‐dependent vasorelaxation and an endothelium‐independent constriction (Laniyonu & Hollenberg, 1995). In sum, the vascular actions of PAR1 stimulation appear to differ, depending on the arterial bed in which PARs are expressed.

PARs are reported to exert critical roles in regulating vascular function by controlling contraction and by modulating endothelial permeability (Klarenbach et al., 2003; Ramachandran et al., 2012; Tauseef et al., 2008). For example, injection of PAR1‐ and PAR2‐activating peptides caused hypotension and hypertension, respectively, in rats (Cicala et al., 2001). PAR1 and PAR2 activation also evoked endothelium‐independent contraction in coronary arteries (El‐Daly et al., 2014). In various cell lines, PAR1 activation generates an increase in cell activity. In human umbilical vein endothelial cells (HUVECs), for example, PAR1 activation evoked cytosolic Ca^2+^ changes to induce endothelial permeability changes (Amerongen et al., 1998; O'Brien et al., 2000). In a human pulmonary arterial endothelial cell line, PAR1 activation evoked an increase in cytosolic Ca^2+^ concentration and protein kinase Cα activity that induced cytoskeleton reorganization, endothelial cell contraction, and permeability changes (Komarova et al., 2007; Singh et al., 2007). PAR1 was also reported to be linked to β‐arrestin and Wnt signaling in HUVEC‐derived EA.hy926 cells (Soh & Trejo, 2011).

In the present study, PAR2 activation with specific agonists (2fLI and trypsin) elicited both Ca^2+^ responses in endothelial cells and endothelial‐dependent vasodilation. However, activation of PAR1 failed to alter endothelial Ca^2+^ signaling or contraction in intact mesenteric arteries. The reason for the differences in the present findings and previous results obtained using isolated aorta‐derived rings is unclear but may be related either to a difference in the endothelial phenotype expressed in aorta versus mesenteric artery or a requirement for PARs to interact with other receptor activities to modulate signaling pathways (Gieseler et al., 2013). For example, in COS‐7 cell lines, PAR1 and PAR2 receptors, when co‐expressed, responded to thrombin even though each receptor alone did not (O'Brien et al., 2000). PAR1 and PAR2 may also interact by forming heterodimers to mediate the β‐arrestin signaling pathway in cell lines (Lin et al., 2013). However, our experiments show that PAR1 activation does not modulate PAR2 activity. This result suggests limited cross‐talk between PARs to trigger Ca^2+^ release in native endothelial cells. The differences in results may be explained by changes in the behavior of native endothelial cells in intact arteries when compared to cultured endothelial cells. The absence of response to PAR1 activation also raises the possibility that PAR1 activity may generate a cell response without evoking changes in intracellular Ca^2+^. Alternatively, it is possible that thrombin alone is unable to trigger activation of native mesenteric endothelial cells in intact vessels. Thrombin mediates blood clotting by acting in coordination with other coagulation tissue factors when endothelial cells are damaged or dysfunctional (Mann et al., 2003; Minami et al., 2004). This absence of response to thrombin in the mesenteric vessels may ensure that native endothelial cells prevent blood clotting and leukocyte extravasation when small concentrations of circulating thrombin are present. Further investigation is needed to explore the precise role of PAR1 and thrombin in native mesenteric artery‐derived endothelial cells.

Our study revealed significant heterogeneity in the Ca^2+^ responses evoked by PAR2 activation, compared with ACh‐mediated muscarinic receptor activation, both in terms of Ca^2+^ signaling dynamics and in endothelial location. There were two main features of the heterogeneity. First, ACh and 2fLI each evoked activity in different endothelial cell clusters. Approximately 40% of the response to each agonist was derived from cells that were sensitive to only one agonist. The remaining ~60% of cells, responded to PAR2 and muscarinic receptor activation. Presumably expression and distribution of the receptors to subpopulations of endothelial cells accounts for this heterogeneity (Lee et al., 2018; Wilson et al., 2016). The heterogeneity permits the endothelium to process multiple forms of extracellular signals, simultaneously, by spatially segregating various functions to different regions of the endothelium and processing them in parallel (Lee et al., 2018; McCarron et al., 2017, 2019; Wilson et al., 2016). Interestingly, cells that responded to PAR2 and muscarinic receptor activation had an increased Ca^2+^ response and higher frequency in Ca^2+^ oscillations when compared to cells that were activated by either agonist alone. Perhaps these cells act as part of a coordinating “hub” in the endothelial network to link various vascular activities (Lee et al., 2022).

Another aspect of the heterogeneity lay in differences in the kinetics and amplitude of the Ca^2+^ signals evoked by PAR2 and muscarinic receptor activation. There was a delayed occurrence of 2fLI‐ and trypsin‐evoked Ca^2+^ signals when compared to those of ACh. Furthermore, the amplitude of the response increased slowly in those Ca^2+^ signals evoked by 2fLI and trypsin when compared to ACh. The differences in signal presumably links to variations in the intracellular signaling systems.

Here we also show that, PAR2‐evoked vasodilation of precontracted rat mesenteric arteries is dependent on an intact endothelium. When the endothelium was removed, the relaxation was abolished. PAR2 activation also generates a nitric oxide‐dependent arterial and venous dilation in humans (Robin et al., 2003) and endothelium‐dependent and ‐independent responses occurred in mice and rats (Moffatt & Cocks, 1998; Saifeddine et al., 1996; Sobey et al., 1999).

In the present study, PAR2‐evoked Ca^2+^ signals were blocked by a PLC inhibitor, IP_3_R blockers, and by depletion of the internal store. These results suggest that PAR2 activates PLC to generate IP_3_ and activate IP_3_R‐mediated Ca^2+^ release (see also Hollenberg et al., 2008; Kanke et al., 2005; Molino et al., 1998; Paria et al., 2006; Sundivakkam et al., 2013). In other studies, PAR2 has been reported to couple with TRPV4 to induce Ca^2+^ influx and inflammation in the HEK293 cell line (Poole et al., 2013). However, this connection was not observed in the present study. A TRPV4 blocker failed to alter the PAR2 response. These observations indicate PAR2's flexibility and versatility in using various signaling pathways across different tissues.

**Figure 8:**
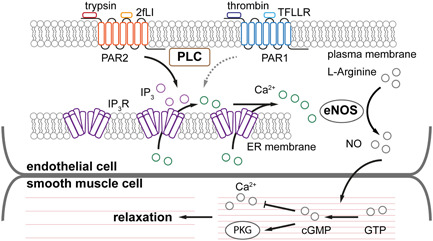
Mechanism of PAR2 mediated endothelial dependent vasodilation. PAR2 activates PLC to produce IP_3_ and activate IP_3_ receptors. Ca^2+^ ions are released from the internal store and trigger further opening of IP_3_ receptors to generate propagating Ca^2+^ waves. Nitric oxide (NO) is produced following Ca^2+^ release from ER and diffuses to the smooth muscle cells (Zhao et al., 2015). PAR1 activation fails to raise Ca^2+^ concentration in native endothelial cells. cGMP, cyclic guanosine monophosphate; IP_3,_ inositol trisphosphate; eNOS, endothelial nitric oxide synthase; ER, endoplasmic reticulum; GTP, guanosine triphosphate; PAR2, protease activated receptor 2; PKG, protein kinase G; PLC, phospholipase C.

PARs play a key role in the interactions that occur between clotting proteases that affect platelets, endothelial cells, and vascular smooth muscle cells to regulate hemostasis, vascular barrier function, vascular contraction, vascular homeostasis, cell adhesion, and inflammatory responses (Leger et al., 2006). Indeed, because inflammatory conditions contribute substantially to the development of cardiovascular diseases, PARs have attracted significant attention since several inflammatory effects are mediated by proteolytic activation of PARs. However, development of a clinical application for PAR agonists and antagonists has been limited. Species and tissue differences in PAR expression, multiple functional roles of the receptors in a wide variety of tissues, and apparently contradictory inflammatory and inflammation‐resolution actions of PAR activation, have limited progress in understanding the physiological effects of PAR activation and identifying a clear‐cut therapeutic target. Upon observing endothelial cell responses in intact mesenteric resistance arteries, we reveal that PAR2 activation triggers cytosolic Ca^2+^ release via IP_3_R to generate global Ca^2+^ waves. PAR2 also mediates endothelial dependent vasodilation in mesenteric arteries (Figure 8). On the other hand, PAR1 activation neither evokes vasodilation, contraction, or changes in cytosolic Ca^2+^ concentration. These findings identify additional complexity in the distribution and function of PAR receptors in the vascular endothelium.

## CONFLICT OF INTEREST STATEMENT

The authors declare no conflict of interest.

## Supporting information

Supporting information.

## References

[jcp30973-bib-0001] Adams, M. N. , Ramachandran, R. , Yau, M. K. , Suen, J. Y. , Fairlie, D. P. , Hollenberg, M. D. , & Hooper, J. D. (2011). Structure, function and pathophysiology of protease activated receptors. Pharmacology & Therapeutics, 130(3), 248–282. 10.1016/j.pharmthera.2011.01.003 21277892

[jcp30973-bib-0002] Alexander, S. P. H. , Christopoulos, A. , Davenport, A. P. , Kelly, E. , Mathie, A. , Peters, J. A. , Veale, E. L. , Armstrong, J. F. , Faccenda, E. , Harding, S. D. , Pawson, A. J. , Sharman, J. L. , Southan, C. , Davies, J. A. , Abbracchio, M. P. , Alexander, W. , Al‐hosaini, K. , Bäck, M. , Beaulieu, J. M. , … Yao, C. (2019). THE CONCISE GUIDE TO PHARMACOLOGY 2019/20: G protein‐coupled receptors. British Journal of Pharmacology, 176(S1), S21–S141. 10.1111/bph.14748 31710717 PMC6844580

[jcp30973-bib-0003] Amerongen, G. P. N. , Draijer, R. , Vermeer, M. A. , & van Hinsbergh, V. W. M. (1998). Transient and prolonged increase in endothelial permeability induced by histamine and thrombin. Circulation Research, 83(11), 1115–1123. 10.1161/01.RES.83.11.1115 9831706

[jcp30973-bib-0004] Anthoni, C. , Russell, J. , Wood, K. C. , Stokes, K. Y. , Vowinkel, T. , Kirchhofer, D. , & Granger, D. N. (2007). Tissue factor: A mediator of inflammatory cell recruitment, tissue injury, and thrombus formation in experimental colitis. Journal of Experimental Medicine, 204(7), 1595–1601. 10.1084/jem.20062354 17562818 PMC2118639

[jcp30973-bib-0005] Aoqui, C. , Chmielewski, S. , Scherer, E. , Eißler, R. , Sollinger, D. , Heid, I. , Braren, R. , Schmaderer, C. , Megens, R. T. , Weber, C. , Heemann, U. , Tschöp, M. , & Baumann, M. (2014). Microvascular dysfunction in the course of metabolic syndrome induced by high‐fat diet. Cardiovascular Diabetology, 13(1), 31. 10.1186/1475-2840-13-31 24490784 PMC3916304

[jcp30973-bib-0006] Austin, K. M. , Covic, L. , & Kuliopulos, A. (2013). Matrix metalloproteases and PAR1 activation. Blood, 121(3), 431–439. 10.1182/blood-2012-09-355958 23086754 PMC3548166

[jcp30973-bib-0007] Boire, A. , Covic, L. , Agarwal, A. , Jacques, S. , Sherifi, S. , & Kuliopulos, A. (2005). PAR1 is a matrix metalloprotease‐1 receptor that promotes invasion and tumorigenesis of breast cancer cells. Cell, 120(3), 303–313. 10.1016/j.cell.2004.12.018 15707890

[jcp30973-bib-0008] Bono, F. , Lamarche, I. , & Herbert, J. M. (1997). Induction of vascular smooth muscle cell growth by selective activation of the proteinase activated receptor‐2 (PAR‐2). Biochemical and Biophysical Research Communications, 241(3), 762–764. 10.1006/bbrc.1997.7847 9434782

[jcp30973-bib-0009] Capers, Q. , Laursen, J. B. , Fukui, T. , Rajagopalan, S. , Mori, I. , Lou, P. , Freeman, B. A. , Berrington, W. R. , Griendling, K. K. , Harrison, D. G. , Runge, M. S. , Alexander, R. W. , & Taylor, W. R. (1997). Vascular thrombin receptor regulation in hypertensive rats. Circulation Research, 80(6), 838–844. 10.1161/01.RES.80.6.838 9168786

[jcp30973-bib-0010] De Ceunynck, K. , Peters, C. G. , Jain, A. , Higgins, S. J. , Aisiku, O. , Fitch‐Tewfik, J. L. , Chaudhry, S. A. , Dockendorff, C. , Parikh, S. M. , Ingber, D. E. , & Flaumenhaft, R. (2018). PAR1 agonists stimulate APC‐like endothelial cytoprotection and confer resistance to thromboinflammatory injury. Proceedings of the National Academy of Sciences, 115(5), E982–E991. 10.1073/pnas.1718600115 PMC5798377 29343648

[jcp30973-bib-0011] Cicala, C. , Morello, S. , Vellecco, V. , Severino, B. , Sorrentino, I. , & Cirino, G. (2003). Basal nitric oxide modulates vascular effects of a peptide activating protease‐activated receptor 2. Cardiovascular Research, 60(2), 431–437. 10.1016/S0008-6363(03)00565-0 14613873

[jcp30973-bib-0012] Cicala, C. , Morello, S. , Santagada, V. , Caliendo, G. , Sorrentino, L. , & Cirino, G. (2001). Pharmacological dissection of vascular effects caused by activation of protease‐activated receptor 1 and 2 in anesthetized rats. The FASEB Journal, 15(8), 1433–1435. 10.1096/fj.00-0633fje 11387248

[jcp30973-bib-0013] Cicala, C. , Pinto, A. , Bucci, M. , Sorrentino, R. , Walker, B. , Harriot, P. , & Cirino, G. (1999). *Protease‐Activated Receptor‐2 Involvement in Hypotension in Normal and Endotoxemic Rats In Vivo*. http://www.circulationaha.org 10.1161/01.cir.99.19.2590 10330393

[jcp30973-bib-0014] Clapham, D. E. (2007). Calcium signaling. Cell, 131(6), 1047–1058. 10.1016/j.cell.2007.11.028 18083096

[jcp30973-bib-0015] Corvera, C. U. , Déry, O. , McConalogue, K. , Böhm, S. K. , Khitin, L. M. , Caughey, G. H. , Payan, D. G. , & Bunnett, N. W. (1997). Mast cell tryptase regulates rat colonic myocytes through proteinase‐activated receptor 2. Journal of Clinical Investigation, 100(6), 1383–1393. 10.1172/JCI119658 9294103 PMC508316

[jcp30973-bib-0016] Coughlin, S. R. (2000). Thrombin signalling and protease‐activated receptors. Nature, 407(6801), 258–264. 10.1038/35025229 11001069

[jcp30973-bib-0017] DeFea, K. A. , Zalevsky, J. , Thoma, M. S. , Déry, O. , Mullins, R. D. , & Bunnett, N. W. (2000). β‐Arrestin‐dependent endocytosis of proteinase‐activated receptor 2 is. The Journal of Cell Biology, 148, 15. 10.1083/jcb.148.6.1267 PMC2174299 10725339

[jcp30973-bib-0018] Echeverri, D. , Montes, F. R. , Cabrera, M. , Galán, A. , & Prieto, A. (2010). Caffeine's vascular mechanisms of action. International Journal of Vascular Medicine, 2010, 1–10. 10.1155/2010/834060 PMC3003984 21188209

[jcp30973-bib-0019] Edelstein, A. , Amodaj, N. , Hoover, K. , Vale, R. , & Stuurman, N. (2010). Computer control of microscopes using µManager. Current Protocols in Molecular Biology, 92(1), 14.20.1–14.20.17. 10.1002/0471142727.mb1420s92 PMC3065365 20890901

[jcp30973-bib-0020] Edelstein, A. D. , Tsuchida, M. A. , Amodaj, N. , Pinkard, H. , Vale, R. D. , & Stuurman, N. (2014). Advanced methods of microscope control using μManager software. Journal of Biological Methods, 1(2), e10. 10.14440/jbm.2014.36 25606571 PMC4297649

[jcp30973-bib-0021] Ehrlich, B. E. , Kaftan, E. , Bezprozvannaya, S. , & Bezprozvanny, I. (1994). The pharmacology of intracellular Ca2+‐release channels. Trends in Pharmacological Sciences, 15(5), 145–149. 10.1016/0165-6147(94)90074-4 7754532

[jcp30973-bib-0022] El‐Daly, M. , Saifeddine, M. , Mihara, K. , Ramachandran, R. , Triggle, C. R. , & Hollenberg, M. D. (2014). Proteinase‐activated receptors 1 and 2 and the regulation of porcine coronary artery contractility: A role for distinct tyrosine kinase pathways. British Journal of Pharmacology, 171(9), 2413–2425. 10.1111/bph.12593 24506284 PMC3997280

[jcp30973-bib-0023] Félétou, M. (2012). The Endothelium. Morgan & Claypool Publishers.

[jcp30973-bib-0024] Garcia, J. G. N. , Patterson, C. , Bahler, C. , Aschner, J. , Hart, C. M. , & English, D. (1993). Thrombin receptor activating peptides induce Ca2+ mobilization, barrier dysfunction, prostaglandin synthesis, and platelet‐derived growth factor mRNA expression in cultured endothelium. Journal of Cellular Physiology, 156(3), 541–549. 10.1002/jcp.1041560313 8360259

[jcp30973-bib-0025] Gieseler, F. , Ungefroren, H. , Settmacher, U. , Hollenberg, M. D. , & Kaufmann, R. (2013). Proteinase‐activated receptors (PARs)—focus on receptor‐receptor‐interactions and their physiological and pathophysiological impact. Cell Communication and Signaling, 11, 86. 10.1186/1478-811X-11-86 24215724 PMC3842752

[jcp30973-bib-0026] Grimsey, N. J. , & Trejo, J. (2016). Integration of endothelial protease‐activated receptor‐1 inflammatory signaling by ubiquitin. Current Opinion in Hematology, 23(3), 274–279. 10.1097/MOH.0000000000000232 26845544 PMC4978167

[jcp30973-bib-0027] Ha, H. S. , Lee, S. E. , Lee, H. S. , Kim, G. H. , Yoon, C. J. , Han, J. S. , Lee, J. Y. , & Sohn, U. D. (2017). The signaling of protease‐activated receptor‐2 activating peptide‐induced contraction in cat esophageal smooth muscle cells. Archives of Pharmacal Research, 40(12), 1443–1454. 10.1007/s12272-017-0975-1 29098568

[jcp30973-bib-0028] Heuberger, D. M. , & Schuepbach, R. A. (2019). Protease‐activated receptors (PARs): Mechanisms of action and potential therapeutic modulators in PAR‐driven inflammatory diseases. Thrombosis Journal, 17(1), 4. 10.1186/s12959-019-0194-8 30976204 PMC6440139

[jcp30973-bib-0029] Hill, C. N. , Hernández‐Cáceres, M. P. , Asencio, C. , Torres, B. , Solis, B. , & Owen, G. I. (2020). Deciphering the role of the coagulation cascade and autophagy in cancer‐related thrombosis and metastasis. Frontiers in Oncology, 10, 2646. 10.3389/fonc.2020.605314 PMC7750537 33365273

[jcp30973-bib-0030] Hill‐Eubanks, D. C. , Werner, M. E. , Heppner, T. J. , & Nelson, M. T. (2011). Calcium signaling in smooth muscle. Cold Spring Harbor Perspectives in Biology, 3(9), a004549. 10.1101/cshperspect.a004549 21709182 PMC3181028

[jcp30973-bib-0031] Höcherl, K. , Gerl, M. , & Schweda, F. (2011). Proteinase‐activated receptors 1 and 2 exert opposite effects on renal renin release. Hypertension, 58(4), 611–618. 10.1161/HYPERTENSIONAHA.111.173229 21859963

[jcp30973-bib-0032] Hollenberg, M.D. , & Houle, S. (2005). Proteinases as hormone‐like signal messengers. Swiss Medical Weekly, 135(29–30), 425–432. 16208579 10.4414/smw.2005.11037

[jcp30973-bib-0033] Hollenberg, M. D. , Renaux, B. , Hyun, E. , Houle, S. , Vergnolle, N. , Saifeddine, M. , & Ramachandran, R. (2008). Derivatized 2‐furoyl‐LIGRLO‐amide, a versatile and selective probe for proteinase‐activated receptor 2: Binding and visualization. Journal of Pharmacology and Experimental Therapeutics, 326(2), 453–462. 10.1124/jpet.108.136432 18477767

[jcp30973-bib-0034] Hollenberg, M. D. , Saifeddine, M. , al‐Ani, B. , & Kawabata, A. (1997). Proteinase‐activated receptors: Structural requirements for activity, receptor cross‐reactivity, and receptor selectivity of receptor‐activating peptides. Canadian Journal of Physiology and Pharmacology, 75(7), 832–841. 9315351

[jcp30973-bib-0035] Kanke, T. , Ishiwata, H. , Kabeya, M. , Saka, M. , Doi, T. , Hattori, Y. , Kawabata, A. , & Plevin, R. (2005). Binding of a highly potent protease‐activated receptor‐2 (PAR2) activating peptide, [3H]2‐furoyl‐LIGRL‐NH2, to human PAR2: Binding of [3H]2‐furoyl‐LIGRL‐NH2to PAR2. British Journal of Pharmacology, 145(2), 255–263. 10.1038/sj.bjp.0706189 15765104 PMC1576136

[jcp30973-bib-0036] Kanke, T. , Macfarlane, S. R. , Seatter, M. J. , Davenport, E. , Paul, A. , McKenzie, R. C. , & Plevin, R. (2001). Proteinase‐activated Receptor‐2‐mediated activation of stress‐activated protein kinases and inhibitory κB kinases in NCTC 2544 keratinocytes. Journal of Biological Chemistry, 276(34), 31657–31666. 10.1074/jbc.M100377200 11413129

[jcp30973-bib-0037] Kawabata, A. , Kuroda, R. , Minami, T. , Kataoka, K. , & Taneda, M. (1998). Increased vascular permeability by a specific agonist of protease‐activated receptor‐2 in rat hindpaw. British Journal of Pharmacology, 125(3), 419–422. 10.1038/sj.bjp.0702063 9806321 PMC1565636

[jcp30973-bib-0038] Klarenbach, S. W. , Chipiuk, A. , Nelson, R. C. , Hollenberg, M. D. , & Murray, A. G. (2003). Differential actions of PAR2 and PAR1 in stimulating human endothelial cell exocytosis and permeability. Circulation Research, 92(3), 272–278. 10.1161/01.RES.0000057386.15390.A3 12595338

[jcp30973-bib-0039] Komarova, Y. A. , Mehta, D. , & Malik, A. B. (2007). Dual regulation of endothelial junctional permeability. Science's STKE, 2007(412), 4122007re8. 10.1126/stke.4122007re8 18000237

[jcp30973-bib-0040] Laniyonu, A. A. , & Hollenberg, M. D. (1995). Vascular actions of thrombin receptor‐derived polypeptides: Structure‐activity profiles for contractile and relaxant effects in rat aorta. British Journal of Pharmacology, 114(8), 1680–1686. 10.1111/j.1476-5381.1995.tb14957.x 7541284 PMC1510399

[jcp30973-bib-0041] Lawton, P. F. , Lee, M. D. , Saunter, C. D. , Girkin, J. M. , McCarron, J. G. , & Wilson, C. (2019). VasoTracker, a low‐cost and open source pressure myograph system for vascular physiology. Frontiers in Physiology, 10, 99. 10.3389/fphys.2019.00099 30846942 PMC6393368

[jcp30973-bib-0042] Lee, M. D. , Buckley, C. , Zhang, X. , Louhivuori, L. , Uhlén, P. , Wilson, C. , & McCarron, J. G. (2022). Small‐world connectivity dictates collective endothelial cell signaling. Proceedings of the National Academy of Sciences, 119(18), e2118927119. 10.1073/pnas.2118927119 PMC9170162 35482920

[jcp30973-bib-0043] Lee, M. D. , Wilson, C. , Saunter, C. D. , Kennedy, C. , Girkin, J. M. , & McCarron, J. G. (2018). Spatially‐structured cell populations process multiple sensory signals in parallel in intact vascular endothelium. Science Signaling, 11(561), eaar4411. 10.1126/scisignal.aar4411 30563865 PMC6420068

[jcp30973-bib-0044] Leger, A. J. , Jacques, S. L. , Badar, J. , Kaneider, N. C. , Derian, C. K. , Andrade‐Gordon, P. , Covic, L. , & Kuliopulos, A. (2006). Blocking the protease‐activated receptor 1‐4 heterodimer in platelet‐mediated thrombosis. Circulation, 113(9), 1244–1254. 10.1161/CIRCULATIONAHA.105.587758 16505172

[jcp30973-bib-0045] Lin, H. , Liu, A. P. , Smith, T. H. , & Trejo, J. (2013). Cofactoring and dimerization of proteinase‐activated receptors. Pharmacological Reviews, 65(4), 1198–1213. 10.1124/pr.111.004747 24064459 PMC3799237

[jcp30973-bib-0046] Mann, K. G. , Butenas, S. , & Brummel, K. (2003). The dynamics of thrombin formation. Arteriosclerosis, Thrombosis, and Vascular Biology, 23(1), 17–25. 10.1161/01.ATV.0000046238.23903.FC 12524220

[jcp30973-bib-0047] McCarron, J. G. , Lee, M. D. , & Wilson, C. (2017). The endothelium solves problems that endothelial cells do not know exist. Trends In Pharmacological Sciences, 38(4), 322–338. 10.1016/j.tips.2017.01.008 28214012 PMC5381697

[jcp30973-bib-0048] McCarron, J. G. , Wilson, C. , Heathcote, H. R. , Zhang, X. , Buckley, C. , & Lee, M. D. (2019). Heterogeneity and emergent behaviour in the vascular endothelium. Current Opinion in Pharmacology, 45, 23–32. 10.1016/j.coph.2019.03.008 31005824 PMC6700393

[jcp30973-bib-0049] McGuire, J. J. , Saifeddine, M. , Triggle, C. R. , Sun, K. , & Hollenberg, M. D. (2004). 2‐Furoyl‐LIGRLO‐amide: A potent and selective proteinase‐activated receptor 2 agonist. Journal of Pharmacology and Experimental Therapeutics, 309(3), 1124–1131. 10.1124/jpet.103.064584 14976230

[jcp30973-bib-0050] Minami, T. , Sugiyama, A. , Wu, S.‐Q. , Abid, R. , Kodama, T. , & Aird, W. C. (2004). Thrombin and phenotypic modulation of the endothelium. Arteriosclerosis, Thrombosis, and Vascular Biology, 24(1), 41–53. 10.1161/01.ATV.0000099880.09014.7D 14551154

[jcp30973-bib-0051] Moffatt, J. D. , & Cocks, T. M. (1998). Endothelium‐dependent and ‐independent responses to protease‐activated receptor‐2 (PAR‐2) activation in mouse isolated renal arteries: Special report. British Journal of Pharmacology, 125(4), 591–594. 10.1038/sj.bjp.0702157 9831889 PMC1571042

[jcp30973-bib-0052] Molino, M. , Raghunath, P. N. , Kuo, A. , Ahuja, M. , Hoxie, J. A. , Brass, L. F. , & Barnathan, E. S. (1998). Differential expression of functional protease‐activated receptor‐2 (PAR‐2) in human vascular smooth muscle cells. Arteriosclerosis, Thrombosis, and Vascular Biology, 18(5), 825–832. 10.1161/01.ATV.18.5.825 9598843

[jcp30973-bib-0053] Morris, D. R. , Ding, Y. , Ricks, T. K. , Gullapalli, A. , Wolfe, B. L. , & Trejo, J. (2006). Protease‐activated receptor‐2 is essential for factor VIIa and Xa‐induced signaling, migration, and invasion of breast cancer cells. Cancer Research, 66(1), 307–314. 10.1158/0008-5472.CAN-05-1735 16397244

[jcp30973-bib-0054] Mosnier, L. O. , Sinha, R. K. , Burnier, L. , Bouwens, E. A. , & Griffin, J. H. (2012). Biased agonism of protease‐activated receptor 1 by activated protein C caused by noncanonical cleavage at Arg46. Blood, 120(26), 5237–5246. 10.1182/blood-2012-08-452169 23149848 PMC3537315

[jcp30973-bib-0055] Nanevicz, T. , Ishii, M. , Wang, L. , Chen, M. , Chen, J. , Turck, C. W. , Cohen, F. E. , & Coughlin, S. R. (1995). Mechanisms of thrombin receptor agonist specificity. Chimeric receptors and complementary mutations identify an agonist recognition site. Journal of Biological Chemistry, 270(37), 21619–21625. 10.1074/jbc.270.37.21619 7665575

[jcp30973-bib-0056] O'Brien, P. J. , Prevost, N. , Molino, M. , Hollinger, M. K. , Woolkalis, M. J. , Woulfe, D. S. , & Brass, L. F. (2000). Thrombin responses in human endothelial cells. Journal of Biological Chemistry, 275(18), 13502–13509. 10.1074/jbc.275.18.13502 10788464

[jcp30973-bib-0057] O'Loughlin, A. J. , O'sullivan, C. J. , Ravikumar, N. , Friel, A. M. , Elliott, J. T. , & Morrison, J. J. (2005). Effects of thrombin, PAR‐1 activating peptide and a PAR‐1 antagonist on umbilical artery resistance in vitro. Reproductive Biology and Endocrinology: RB&E, 3, 8. 10.1186/1477-7827-3-8 15730558 PMC554978

[jcp30973-bib-0058] Paria, B. C. , Bair, A. M. , Xue, J. , Yu, Y. , Malik, A. B. , & Tiruppathi, C. (2006). Ca2+ influx induced by protease‐activated receptor‐1 activates a feed‐forward mechanism of TRPC1 expression via nuclear factor‐κB activation in endothelial cells. Journal of Biological Chemistry, 281(30), 20715–20727. 10.1074/jbc.M600722200 16709572

[jcp30973-bib-0059] Paul, M. , Murphy, S. F. , Hall, C. , Schaeffer, A. J. , & Thumbikat, P. (2019). Protease‐activated receptor 2 activates CRAC‐mediated Ca2+ influx to cause prostate smooth muscle contraction. FASEB BioAdvances, 1(4), 255–264. 10.1096/fba.2018-00024 31198907 PMC6563600

[jcp30973-bib-0060] Pawlinski, R. , Pedersen, B. , Schabbauer, G. , Tencati, M. , Holscher, T. , Boisvert, W. , Andrade‐Gordon, P. , Frank, R. D. , & Mackman, N. (2004). Role of tissue factor and protease‐activated receptors in a mouse model of endotoxemia. Blood, 103(4), 1342–1347. 10.1182/blood-2003-09-3051 14576054 PMC2860856

[jcp30973-bib-0061] Poole, D. P. , Amadesi, S. , Veldhuis, N. A. , Abogadie, F. C. , Lieu, T. , Darby, W. , Liedtke, W. , Lew, M. J. , McIntyre, P. , & Bunnett, N. W. (2013). Protease‐activated receptor 2 (PAR2) protein and transient receptor potential vanilloid 4 (TRPV4) protein coupling is required for sustained inflammatory signaling. Journal of Biological Chemistry, 288(8), 5790–5802. 10.1074/jbc.M112.438184 23288842 PMC3581372

[jcp30973-bib-0062] Ramachandran, R. , Noorbakhsh, F. , Defea, K. , & Hollenberg, M. D. (2012). Targeting proteinase‐activated receptors: Therapeutic potential and challenges. Nature Reviews Drug Discovery, 11(1), 69–86. 10.1038/nrd3615 22212680

[jcp30973-bib-0082] Robin, J. , Kharbanda, R. , Mclean, P. , Campbell, R. , & Vallance, P . (2003). Protease‐activated receptor 2–mediated vasodilatation in humans in vivo. Circulation, 107(7), 954–959. 10.1161/01.cir.0000050620.37260.75 12600906

[jcp30973-bib-0063] Rothmeier, A. S. , & Ruf, W. (2012). Protease‐activated receptor 2 signaling in inflammation. *Semin Immunopathol*, *34*(1), 133‐149. 10.1007/s00281-011-0289-1 21971685

[jcp30973-bib-0064] Saifeddine, M. , Al‐Ani, B. , Cheng, C.‐H. , Wang, L. , & Hollenberg, M. D. (1996). Rat proteinase‐activated receptor‐2 (PAR‐2): CDNA sequence and activity of receptor‐derived peptides in gastric and vascular tissue. British Journal of Pharmacology, 118(3), 521–530. 10.1111/j.1476-5381.1996.tb15433.x 8762073 PMC1909734

[jcp30973-bib-0065] Saleem, H. , Tovey, S. C. , Molinski, T. F. , & Taylor, C. W. (2014). Interactions of antagonists with subtypes of inositol 1,4,5‐trisphosphate (IP3) receptor. British Journal of Pharmacology, 171(13), 3298–3312. 10.1111/bph.12685 24628114 PMC4080982

[jcp30973-bib-0066] Seeley, S. , Covic, L. , Jacques, S. L. , Sudmeier, J. , Baleja, J. D. , & Kuliopulos, A. (2003). Structural basis for thrombin activation of a protease‐activated receptor: Inhibition of intramolecular liganding. Chemistry & Biology, 10(11), 1033–1041. 10.1016/j.chembiol.2003.10.014 14652070

[jcp30973-bib-0067] Singh, I. , Knezevic, N. , Ahmmed, G. U. , Kini, V. , Malik, A. B. , & Mehta, D. (2007). Gαq‐TRPC6‐mediated Ca2+ entry induces RhoA activation and resultant endothelial cell shape change in response to thrombin. Journal of Biological Chemistry, 282(11), 7833–7843. 10.1074/jbc.M608288200 17197445

[jcp30973-bib-0068] Sobey, C. G. , Moffatt, J. D. , & Cocks, T. M. (1999). Evidence for selective effects of chronic hypertension on cerebral artery vasodilatation to protease‐activated receptor‐2 activation. Stroke, 30(9), 1933–1941. 10.1161/01.STR.30.9.1933 10471447

[jcp30973-bib-0069] Soh, U. J. K. , & Trejo, J. (2011). Activated protein C promotes protease‐activated receptor‐1 cytoprotective signaling through β‐arrestin and dishevelled‐2 scaffolds. Proceedings of the National Academy of Sciences, 108(50), E1372–E1380. 10.1073/pnas.1112482108 PMC3250136 22106258

[jcp30973-bib-0070] Sundivakkam, P. C. , Natarajan, V. , Malik, A. B. , & Tiruppathi, C. (2013). Store‐operated Ca2+ entry (SOCE) induced by protease‐activated receptor‐1 mediates STIM1 protein phosphorylation to inhibit SOCE in endothelial cells through AMP‐activated protein kinase and p38β mitogen‐activated protein kinase. Journal of Biological Chemistry, 288(23), 17030–17041. 10.1074/jbc.M112.411272 23625915 PMC3675634

[jcp30973-bib-0071] Tauseef, M. , Kini, V. , Knezevic, N. , Brannan, M. , Ramchandaran, R. , Fyrst, H. , Saba, J. , Vogel, S. M. , Malik, A. B. , & Mehta, D. (2008). Activation of sphingosine kinase‐1 reverses the increase in lung vascular permeability through sphingosine‐1‐phosphate receptor signaling in endothelial cells. Circulation Research, 103(10), 1164–1172. 10.1161/01.RES.0000338501.84810.51 18849324 PMC2708004

[jcp30973-bib-0072] Trottier, G. , Hollenberg, M. , Wang, X. , Gui, Y. , Loutzenhiser, K. , & Loutzenhiser, R. (2002). PAR‐2 elicits afferent arteriolar vasodilation by NO‐dependent and NO‐independent actions. American Journal of Physiology‐Renal Physiology, 282(5), F891–F897. 10.1152/ajprenal.00233.2001 11934700

[jcp30973-bib-0073] Vassallo, R. R. , Kieber‐Emmons, T. , Cichowski, K. , & Brass, L. F. (1992). Structure‐function relationships in the activation of platelet thrombin receptors by receptor‐derived peptides. Journal of Biological Chemistry, 267(9), 6081–6085. 10.1016/S0021-9258(18)42664-6 1313429

[jcp30973-bib-0074] Villari, A. , Giurdanella, G. , Bucolo, C. , Drago, F. , & Salomone, S. (2017). Apixaban enhances vasodilatation mediated by protease‐activated receptor 2 in isolated rat arteries. Frontiers in Pharmacology, 8, 480. 10.3389/fphar.2017.00480 28769809 PMC5513931

[jcp30973-bib-0075] Vu, T.‐K. H. , Hung, D. T. , Wheaton, V. I. , & Coughlin, S. R. (1991). Molecular cloning of a functional thrombin receptor reveals a novel proteolytic mechanism of receptor activation. Cell, 64(6), 1057–1068. 10.1016/0092-8674(91)90261-V 1672265

[jcp30973-bib-0076] Wilson, C. , Lee, M. D. , & McCarron, J. G. (2016). Acetylcholine released by endothelial cells facilitates flow‐mediated dilatation. Journal of Physiology, 594(24), 7267–7307. 10.1113/JP272927 27730645 PMC5157078

[jcp30973-bib-0077] Wilson, C. , Saunter, C. D. , Girkin, J. M. , & McCarron, J. G. (2016). Clusters of specialized detector cells provide sensitive and high fidelity receptor signaling in the intact endothelium. The FASEB Journal, 30(5), 2000–2013. 10.1096/fj.201500090 26873937 PMC4836367

[jcp30973-bib-0078] Zhang, P. , Covic, L. , & Kuliopulos, A. (2013). Protease‐activated receptors. In A. D. Michelson (Ed.), Platelets (pp. 249–259). Academic Press. 10.1016/B978-0-12-387837-3.00013-4

[jcp30973-bib-0079] Zhang, X. , Lee, M. D. , Wilson, C. , & McCarron, J. G. (2019). Hydrogen peroxide depolarizes mitochondria and inhibits IP3‐evoked Ca2+ release in the endothelium of intact arteries. Cell Calcium, 84, 102108. 10.1016/j.ceca.2019.102108 31715384 PMC6891240

[jcp30973-bib-0080] Zhao, Y. , Vanhoutte, P. M. , & Leung, S. W. S. (2015). Vascular nitric oxide: Beyond eNOS. Journal of Pharmacological Sciences, 129(2), 83–94. 10.1016/j.jphs.2015.09.002 26499181

