# Supplementary material for: Endothelial PAR2 activation evokes resistance artery relaxation
Source: J Cell Physiol. 2023 Feb 15;238(4):776–89. doi: 10.1002/jcp.30973 (PMC10952239; doi:10.1002/jcp.30973)
Supplement: Supplementary file 1 — Supporting information. [file JCP-238-776-s001.docx]

**SUPPLEMENTARY MATERIAL**


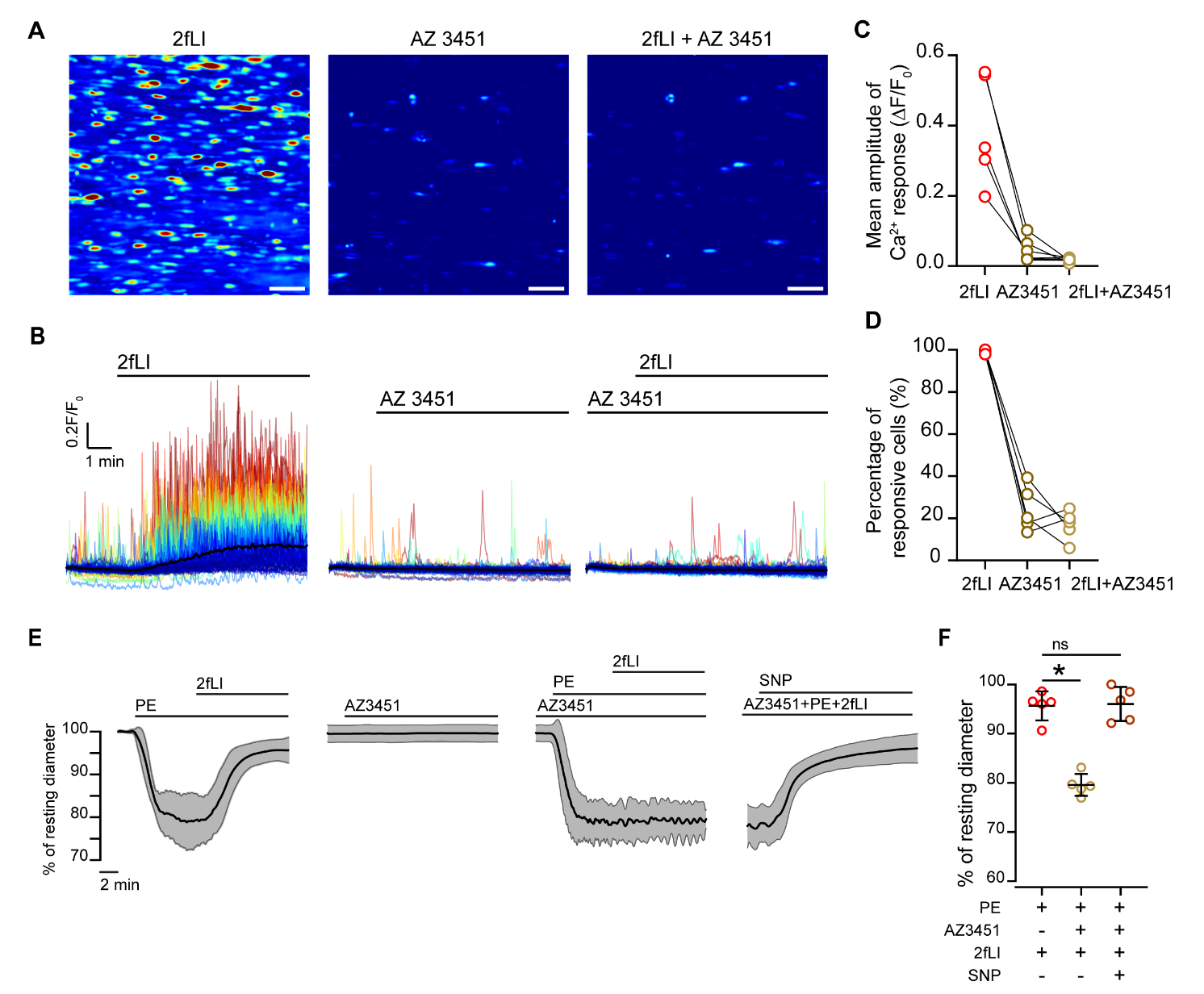


**Figure S1 - PAR2 evoked Ca^2+^ signals and vasodilation are blocked by a PAR2 inhibitor.** (A) Heat map images showing maximum intensity projections (∆F/F_0_) of Ca^2+^ signals evoked by 2fLI, the PAR2 receptor blocker AZ 3451 and 2fLI with AZ 3451. Scale bar= 50 µm. (B) Coloured overlaid Ca^2+^ signalling traces from each cell in (A). Mean peak amplitude of Ca^2+^ signalling (C) and percentage of responsive cells (D) evoked by 2fLI, AZ 3451 and 2fLI with AZ 3451. (E) Plots of diameter change (%) of arteries in 20min recordings. The solid black line shows the average diameter. The grey filling shows the standard deviation (SD) from 5 biological replicates in each experiment. (F) Summarized data of the percentage of relaxation evoked by 2fLI, 2fLI + AZ 3451 and by sodium nitroprusside (SNP, 100 µM). (E) For summary data (C, D, F), *n*=5, **p*<0.05.

**
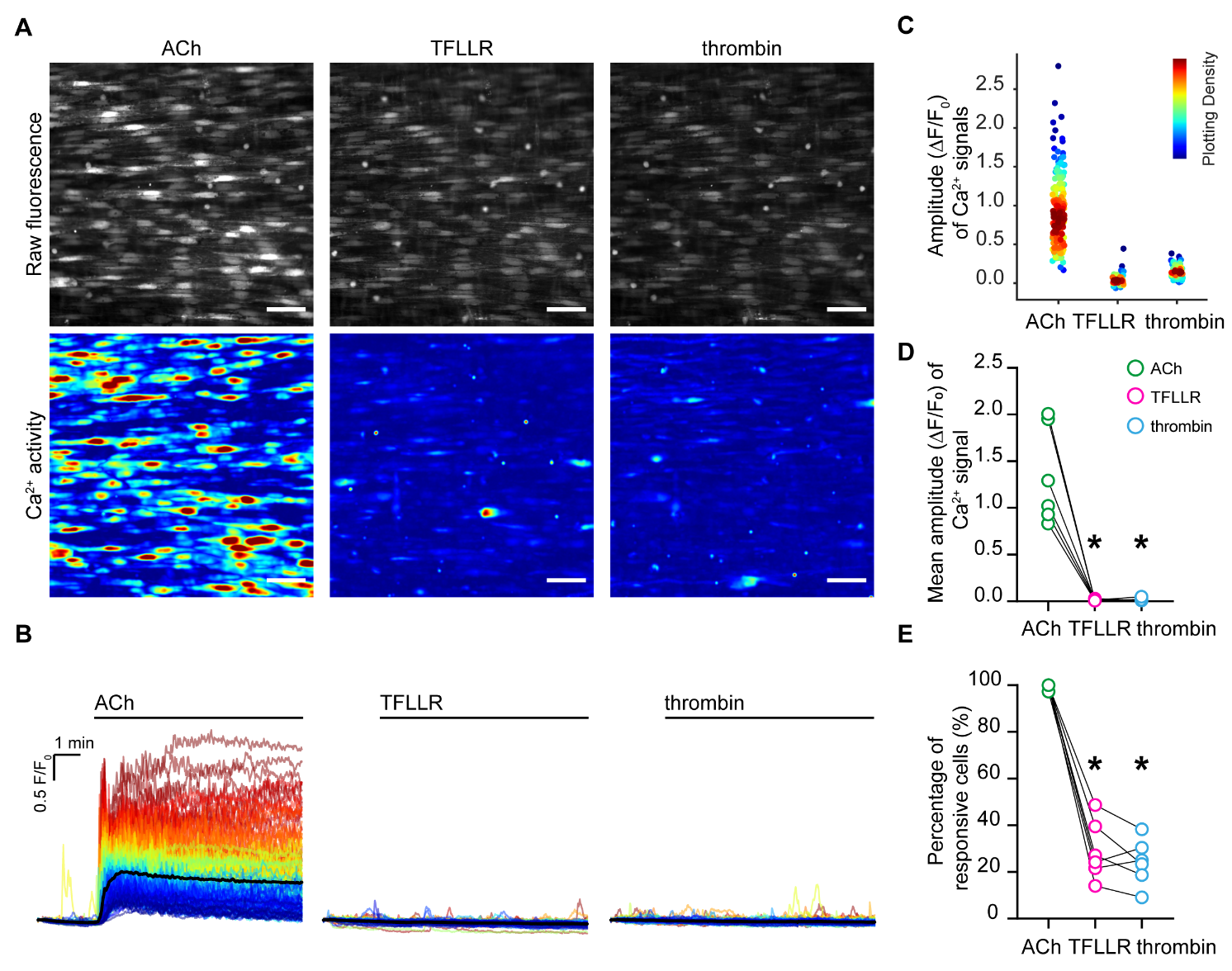
Figure S2 - PAR1 does not generate Ca^2+^ signals in intact mesenteric endothelial cells.** (A) Raw fluorescence (top) and heat maps showing maximum intensity (∆F/F_0_) projections (bottom) of Ca^2+^ signals evoked by ACh (100 nM), the PAR1 activating peptide, TFLLR (10 µM) and the PAR1 agonist thrombin (0.2 U ml^-1^) during 10 min recordings. Scale bar = 50 µm. (B) Corresponding coloured overlaid Ca^2+^ signalling traces from each cell in (A) according to the intensity of signals (F/F_0_, from blue, low to red, high). The bold black line shows the averaged Ca^2+^ signals. (C) Density plot of the peak value of Ca^2+^ signals from each cell in (A). Individual data points have been coloured (from blue, low to red, high) according to the density of particular values. (D) Summarized mean peak amplitude of Ca^2+^ signals evoked by ACh, TFLLR and thrombin. (E) Summarized mean percentage of responsive cells to ACh, TFLLR and thrombin in all cells. For all summary data (D, E), *n*=6, * *p*<0.05.

**
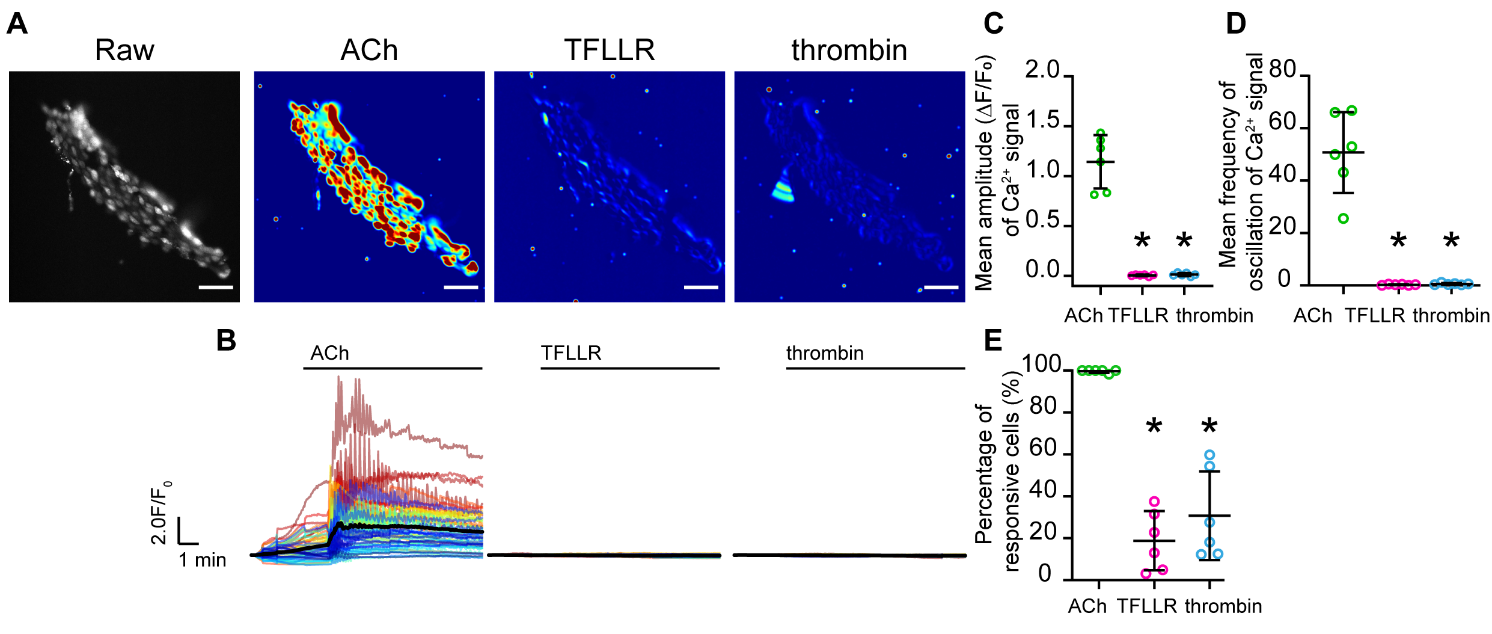
**

**Figure S3 - Endothelial patches did not respond to PAR1 agonists**. (A) Raw fluorescence and heat map of maximum intensity (∆F/F_0_) of Ca^2+^ signals evoked by each agonist during 10 min recordings in endothelial patches. Scale bar = 50 µm. (B) Coloured overlaid Ca^2+^ signalling traces from each cell in (A) plotted according to the intensity of signals (F/F_0_, from blue, low to red, high). The bold black shows the averaged Ca^2+^ signals. Summarized data of the mean peak amplitude (C), frequency (D) of Ca^2+^ signalling and percentage of responsive cells (E) to each agonists. For all summary data (C, D, E), *n*=6, * *p*<0.05.

**­­**

**
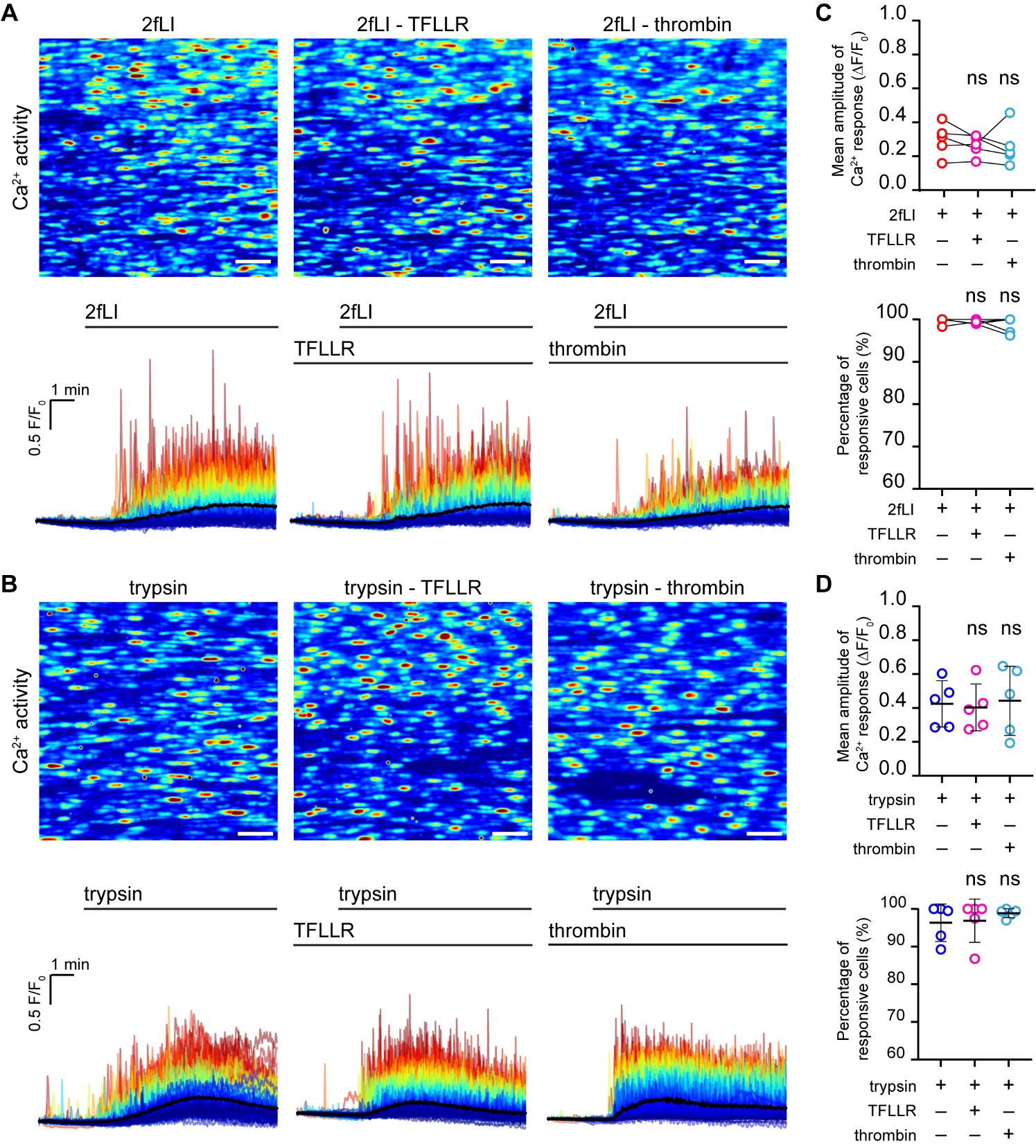
**

**Figure S4. Absence of PAR2 and PAR1 cross-talk in regulating Ca^2+^ signalling in endothelial cells.** (A-B) Top, heat map images showing (A) 2fLI and (B) trypsin-evoked Ca^2+^ activity in the absence and presence of PAR1 activation with TFLLR (10 µM) or thrombin (0.2 U ml^-1^). Bottom, overlaid Ca^2+^ signals of all cells. In trypsin experiments, a new preparation from the same animal was used for each condition. (C-D) Summary data of 2fLI (C) and trypsin (D) evoked Ca^2+^ responses. For all summary data *n*=5, **p*<0.05.


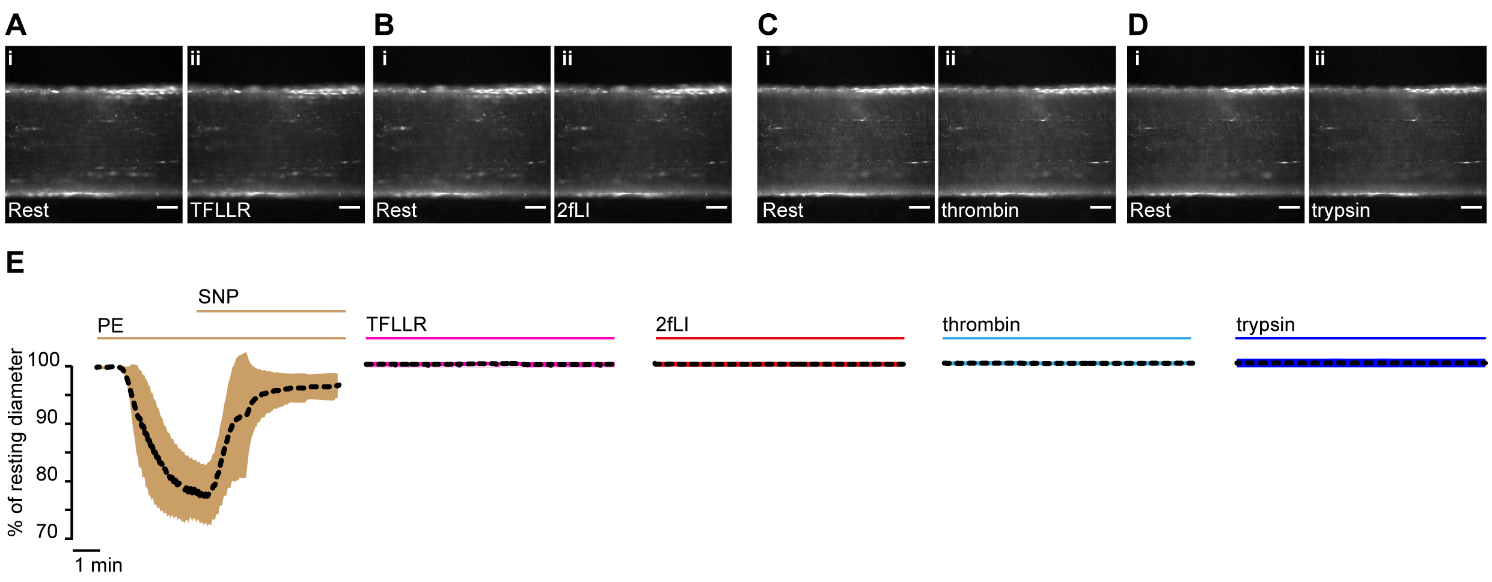


**Figure S5 - PAR1 and PAR2 do not evoke contraction in endothelial denuded arteries.** (A – D) Raw images of the same en face mesenteric artery exhibiting the change of perimeter by TFLLR (10 µM) (A), 2fLI (5 µM) (B), thrombin (0.2 U ml^-1^) (C) and trypsin (2 U ml^-1^) (D). (E) Plots of diameter change (%) to resting status in 10 min recordings. The dashed black line shows the average (percentage) diameter. Coloured filling represents the standard deviation (SD) from 5 different individuals.
